# Supplementary material for: Machine-guided design of cell-type-targeting cis-regulatory elements
Source: Nature. 2024 Oct 23;634(8036):1211–20. doi: 10.1038/s41586-024-08070-z (PMC11525185; doi:10.1038/s41586-024-08070-z)
Supplement: Supplementary file 1 — Supplementary Notes, Supplementary Figs. 1–22 and descriptions for the headers used in Supplementary Tables 1–3, 5–10 and 12. Supplementary Notes include an assessment of model hyperparameter settings, sequence content and diversity, experimental reproducibility of MPRA across projects, and validation of contribution scores for model interpretation. [file 41586_2024_8070_MOESM1_ESM.pdf]

nature portfolio

https://doi.org/10.1038/s41586-024-08070-z

Supplementary information

Machine-guided design of cell-type-targeting *cis*-regulatory elements

In the format provided by the authors and unedited

Supplementary Note 1: Assessment of model hyperparameter settings

Bayesian Optimization (BO) is a technique used to iteratively propose arguments to a black-box function to minimize the output and is commonly used to select hyperparameters for deep-learning algorithms. Malinois was developed using BO over subsets of hyperparameters, leaving the possibility for additional performance gains by joint optimization of all hyperparameters. We conducted a comprehensive BO to evaluate this possibility. BO finds reasonable hyperparameter settings within 100 proposals and additional adjustments produce models with only incremental improvements in performance (Supplementary Note 1 - Figure 1a, Supplementary Table 3). We observed several design choices that impacted the model including the use of transfer learning from Basset. Initializing weights using Basset, a model of chromatin accessibility, is more effective than random initialization (Supplementary Note 1 - Figure 1b). Duplicating and augmenting the training data by taking the reverse complements of the input sequences improves predictions (Supplementary Note 1 - Figure 1c). Using branched linear layers in place of fully connected layers in the final layers of the model can produce some of the highest performing individual models but does not result in a dramatic overall improvement and introduces substantial variance likely due to the lack of transfer learning at these new layers (Supplementary Note 1 - Figure 1d). Notably, transfer learning does not negatively impact generalization accuracy on predictions for synthetic elements (Supplementary Note 1 - Figure 2). Randomly initialized models that did not deploy transfer-learning from chromatin accessibility performed well in practice, though were not favored during Bayesian Optimization of hyperparameters (Pearson's r 0.88-0.89; Spearman's ρ 0.81-0.83). Despite the incomplete BO of hyperparameters when training Malinois, it is only slightly outperformed by the top configurations from this comprehensive BO experiment (Malinois: Pearson's r = 0.877-0.886; Top BayesOpt model: Pearson's r = 0.880-0.890).

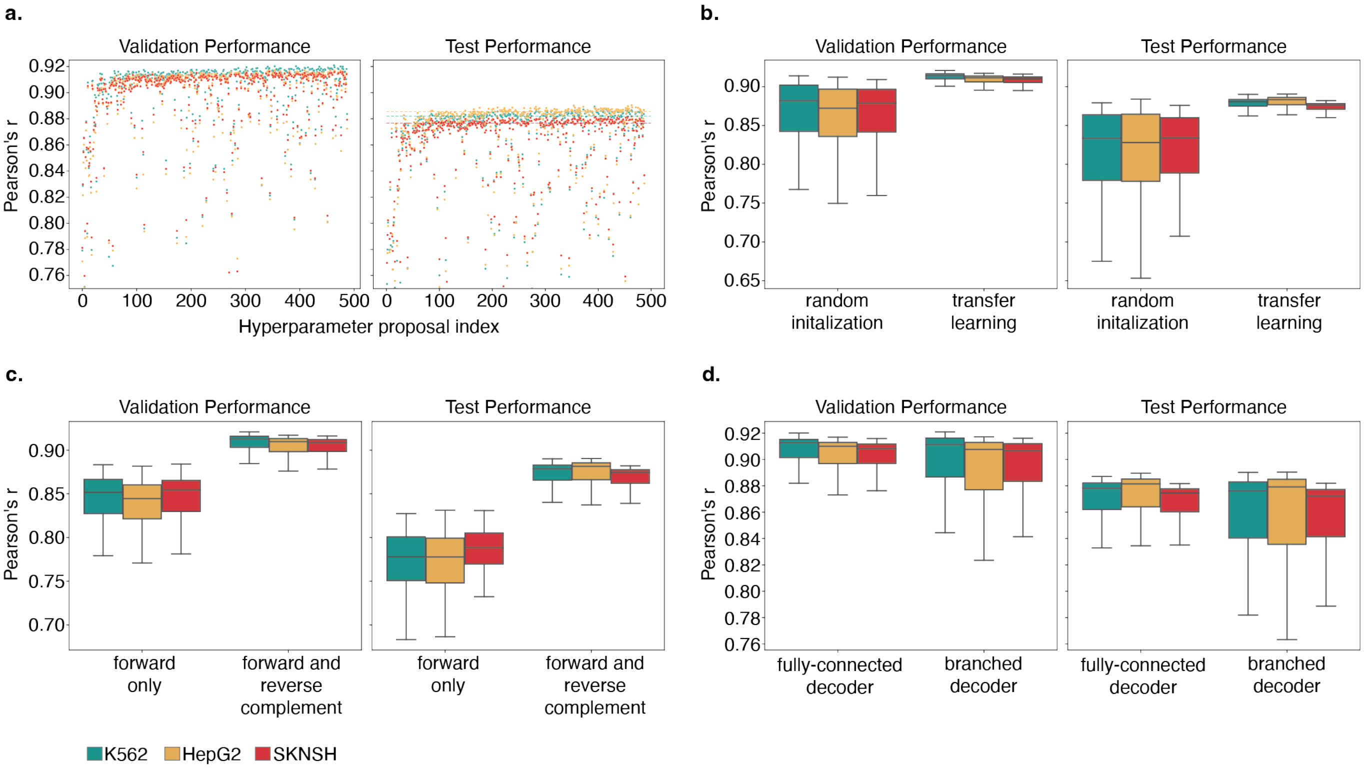

Supplementary Note 1 - Figure 1. Bayesian optimization effectively finds reasonable hyperparameter settings. (a) Validation and test set performance of models from hyperparameter proposals picked by Bayesian Optimization, in order. Dotted lines indicate test set performance of Malinois (n = 492). (b) Transfer learning by initializing weights from Basset results in less variation and overall improvement in training outcomes (n=360 and 132 random initialization and transfer learning models, respectively). (c) Duplicating and augmenting the training data by taking the reverse compliments of the input sequences improves modeling accuracy (n=450 and 42 models trained with and without data augmentation, respectively). (d) Replacing fully-connected layers in the decoder segment of CNNs increases variance in fitted model performance, although the top performing branched decoder models show improvement comparatively (n=280 and 212 branched and fully connected decoder models, respectively). (a)-(d) Pairs of subpanels in each panel share a y-axis. (b)-(d) Boxes demarcate the 25th, 50th, and 75th percentile values, while whiskers indicate the outermost point within 1.5 times the interquartile range from the edges of the boxes.

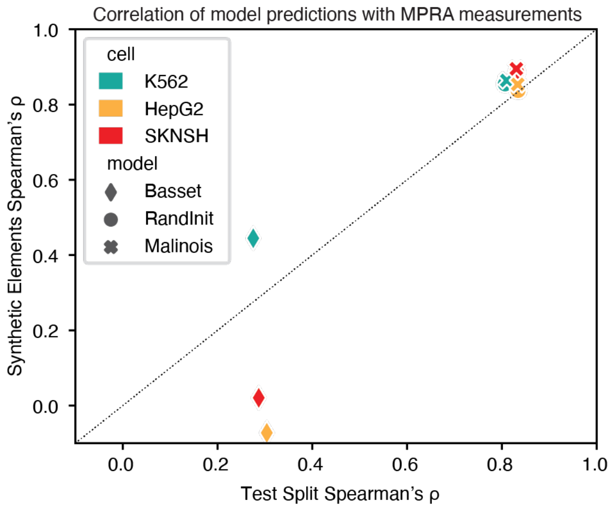

Supplementary Note 1 - Figure 2. Assessment of impacts of transfer learning on generalizability. (**c**) A scatter plot summarizing the predictive performance of the parent Bassest model used for transfer learning, a randomly initialized model fitted to MPRA data without transfer learning, and Malinois. Performance is measured using the test split (oligos derived from chr7 and chr13; x-axis) and synthetic elements (y-axis) to calculate correlation with empirical MPRA measurements. Correlations are calculated separately for cell type. Note, x-shaped markers are overlapping o-shaped markers for each cell type.

Supplementary Note 2: Sequence content and diversity

<u>Sequence penalties alter motif content</u>

Over five iterative rounds, we generated a total of 15,000 'synthetic-penalized' CREs, with 1,000 sequences per round per cell type, while penalizing the top motifs from the preceding rounds in each iteration (**Supplementary Note 2 - Figure 1a**, **Supplementary Table 5, Methods**). We observed successful reduction in initially enriched motifs and a simultaneous increase in motifs underutilized in earlier rounds (**Supplementary Note 2 - Figure 1b**), diversifying the motif content of CODA-proposed sequences for experimental evaluation.

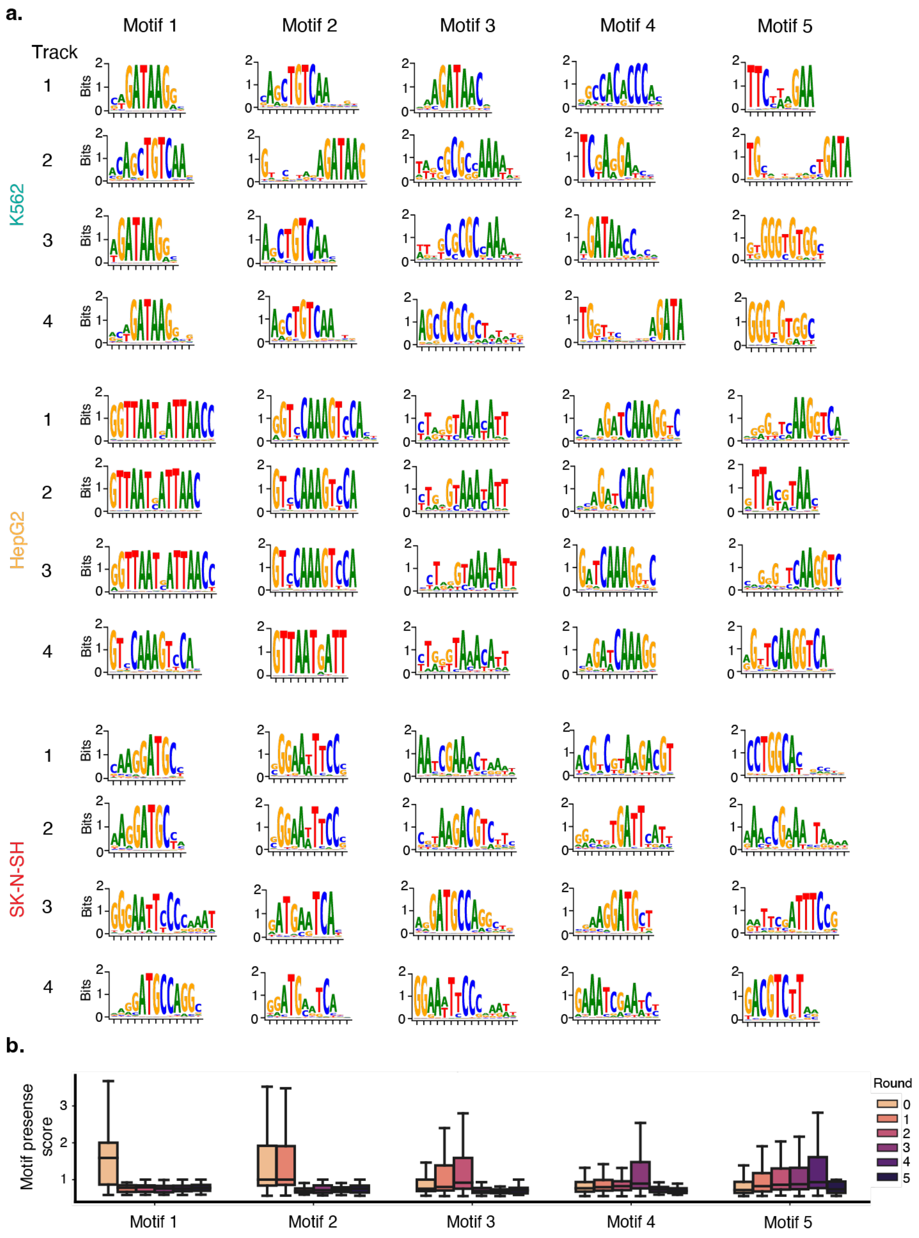

Supplementary Note 2 - Figure 1. Motif match scores during penalization. (**a**) Motifs can be depleted from Fast SeqProp-generated sequences using motif penalization. Motif numbers on the *x*-axis correspond to the first round in which their matches are penalized during Fast SeqProp, as they were the top match from the previous round. For each target cell type, four independent tracks of penalization were carried out (**Methods**) to account for potential enrichment effects of the random initialization when generating sequences. (**b**) Underrepresented motifs are progressively enriched as preferred alternatives are depleted. Box plots capture distribution of motif matches across sequences produced in each round of penalized generation, n=4,000 for round 0, n=3,000 for all other rounds. Motif number labels on the *x*-axis correspond to the round in which their penalization was first introduced during Fast SeqProp optimization. Motifs are specifically depleted in rounds where they are introduced into the penalty calculation, but can gradually rise during preceding rounds. In the *y*-axis, the motif-presence score of each motif is calculated by summing all the motif-match scores that

pass a score threshold in a sequence, and dividing the sum by the score of the motif consensus sequence. Boxes demarcate the 25th, 50th, and 75th percentile values, while whiskers indicate the outermost point within 1.5 times the interquartile range from the edges of the boxes.

All methods generated diverse sequences

To assess the diversity of sequences generated by each method, we first quantified single-nucleotide similarity by calculating the average Levenshtein distance of each sequence to its 4 nearest neighbors within the corresponding design group and repeated this process for human promoters and shuffled sequences from the library as controls (**Supplementary Note 2 - Figure 2a**). DHS-natural, and non-repetitive Malinois-natural sequences were respectively 1.2%, and 11.8% closer to neighbors than shuffled controls. Depending on the generative algorithm, non-penalized synthetic sequences were 0.57%-2.9% closer to neighbors. Interestingly, synthetic-penalized sequences were on average 0.45%-0.89% further away from their 4 nearest neighbors than shuffled controls, with distances increasing during successive penalization rounds (Spearman's ρ=0.73 $p<10^{-300}$). In contrast, promoters were 8.9% closer to neighbors than shuffled controls, implying that synthetic sequences are substantially more diverse than promoters. As a more stringent assessment of diversity that can capture reuse of individual sequence motifs, we also quantified the average distance of 7-mer content to the 4 nearest neighbors for all oligos. On average, non-repetitive natural sequences selected by DHS and Malinois were 3.0% and 24.4% closer to their nearest neighbors, respectively, than shuffled sequences. Synthetic sequence pairs showed median levels of 7-mer diversity in between groups of natural sequences, being on average 3.6%-7.2% closer to nearest neighbors than shuffled sequences. Motif penalization significantly reduced neighbor closeness from 6.5% to 0.82% relative to shuffled controls (Spearman's ρ=0.75, $p<10^{-300}$, **Supplementary Note 2 - Figure 2b**). On the other hand, despite the modest reductions compared to shuffle sequences, all groups except Malinois-natural showed less 7-mer similarity than promoters (on average 9.7% closer to nearest neighbors than shuffled sequences), showing synthetic sequences provide a diverse collection of CREs. Finally, embedding the 4-mer content of the sequences into two-dimensions using UMAP we observed synthetic elements separated by target cell type and from natural elements (**Supplementary Note 2 - Figure 3a-i**) supporting the observation that the synthetic sequences are distinct from sequences found in the human genome as measured by homology search (**Methods**).

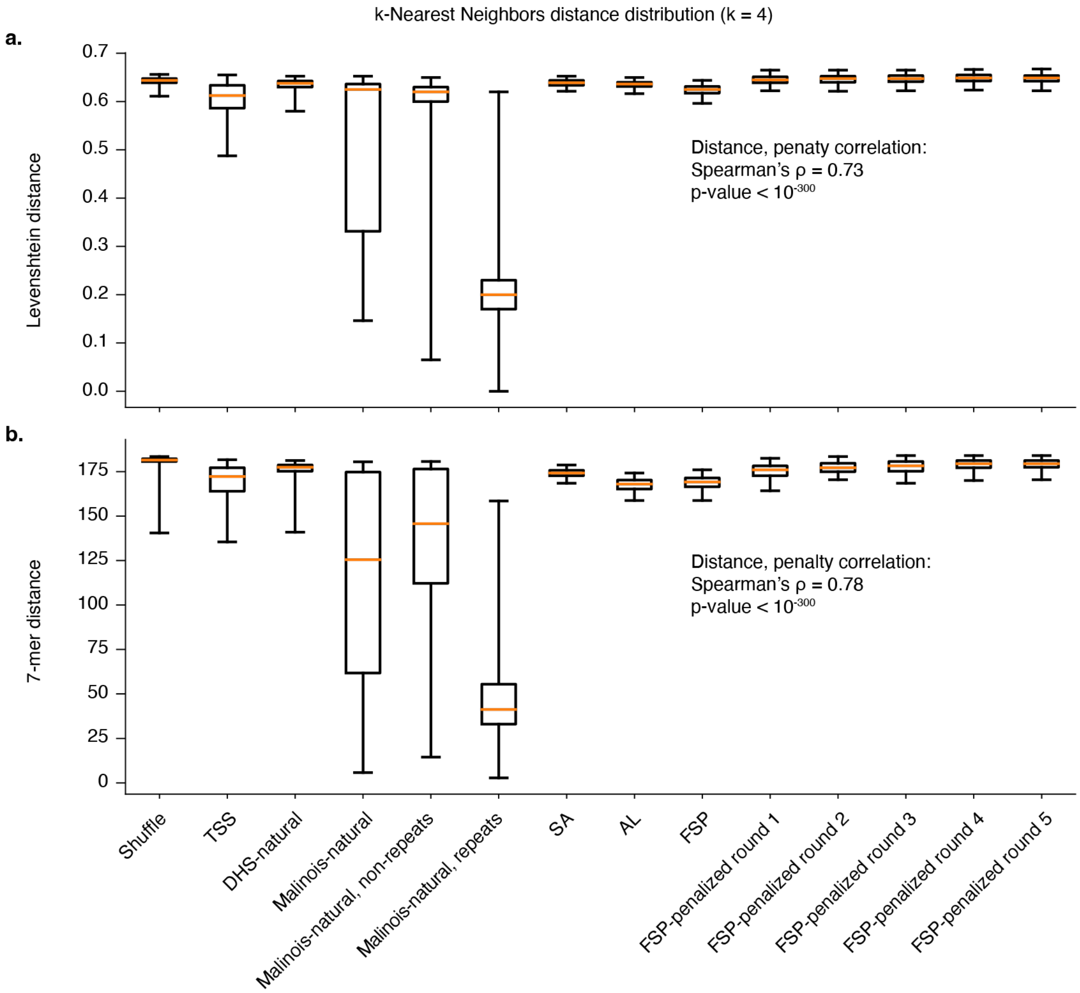

Supplementary Note 2 - Figure 2. K-mer and Hamming distance. (**a**) Algorithms for model-guided sequence designs produce diverse, non-degenerate candidate CREs. Box plot displays the distribution of average Levenshtein distance to 4 nearest neighbors for sequences in categories indicated on the *x*-axis. As a control, we randomly selected 4000 shuffled sequences from the candidate CRE library, and 19381 promoter sequences extracted from RefGene by taking the 200 nucleotides upstream of (strand aware) TSS annotations for mRNAs. Malinois-natural results are plotted on aggregate, only using non-repeat element matched sequences, and repeat element matched sequences. Spearman's correlation coefficient was calculated between penalization round number (starting at zero) and average Hamming distances to 4 nearest neighbors. Boxes demarcate the 25th, 50th, and 75th percentile values, while whiskers indicate the 1st and 99th percentile values. (**b**) Algorithms for model-guided sequence designs produce sequences with diverse, non-redundant 7-mer usage. Plot is the same as **a** except it displays average L1 distances of 7-mer content between sequences and 4 nearest neighbors, divided by 2. Boxes demarcate the 25th, 50th, and 75th percentile values, while whiskers indicate the 1st and 99th percentile values. (a)-(b) Left-to-right, for each box n=4,000; 19,381; 12,000; 12,000; 9,255; 2,745; 12,000; 12,000; 12,000; 3,000; 3,000; 3,000; 3,000; 3,000.

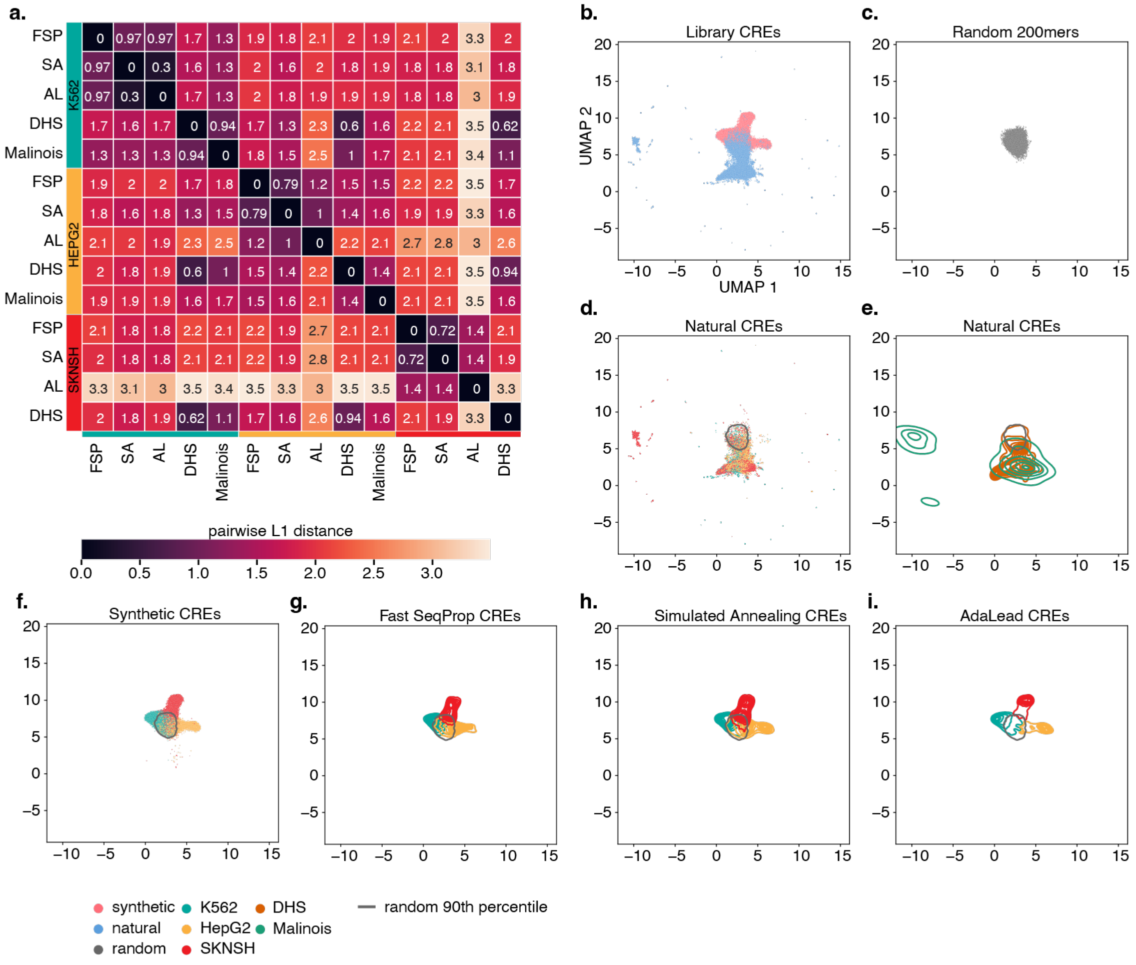

Supplementary Note 2 - Figure 3. Variation in 4-mer content between natural and synthetic cell type specific elements. (**a**) L1 distance between groups of designed CREs based on marginalized 4-mer frequencies in each group. (**b**) UMAP embedding of all non-penalized CREs in the designed cell type specific sequence element library colored by synthetic (pink) or natural (blue) provenance. (**c**) 12,000 random 200-mers embedded in the same UMAP as (a). (**d**) The subset of points in (a) that are natural CREs selected to be cell type specific based on DHS or Malinois predictions, colored by target cell type. (**e**) A kernel density estimate from the natural CREs in (d) but recolored by if the element was selected using DHS (orange) or Malinois (green). (**f**) The subset of points in (a) that are synthetic CREs, colored by target cell type. (**g**) A kernel density estimate from synthetic CREs designed by Fast SeqProp, colored by target cell type. (**h**) Same as (g) except from CREs designed by Simulated annealing. (**i**) Same as (g) except CREs designed by AdaLead. The UMAP region containing 90% of random sequences is indicated by a gray line in (d)-(i).

Lexical analysis of motif content

In linguistics, the number of types refers to the total number of unique words in a given text segment, while the number of tokens refers to the total number of words. The type-token ratio (TTR) represents the degree of lexical variation, the relationship between the number of unique words observed in a segment and their frequencies. In our case, motif instances are treated as words. We observed that synthetic sequences not only contain more motif instances (tokens) per sequence (7 more tokens in median vs natural; $p<10^{-300}$, one-sided Wilcoxon rank-sum test; **Supplementary Note 2 - Figure 4a,b**), but also contain a greater number of different motifs (types) per sequence (2 more types in median vs natural; $p<10^{-300}$, one-sided Wilcoxon rank-sum test). On the other hand, penalized synthetic sequences showed a greater TTR compared to non-penalized ones (median TTR 0.58 vs 0.5 respectively; $p<10^{-300}$, one-sided Wilcoxon rank-sum test; **Supplementary Note 2 - Figure 4c,d**). As these penalized sequences remain highly specific, CODA is able to explore alternative regulatory mechanisms successfully despite increased syntactical constraints posed by penalization.

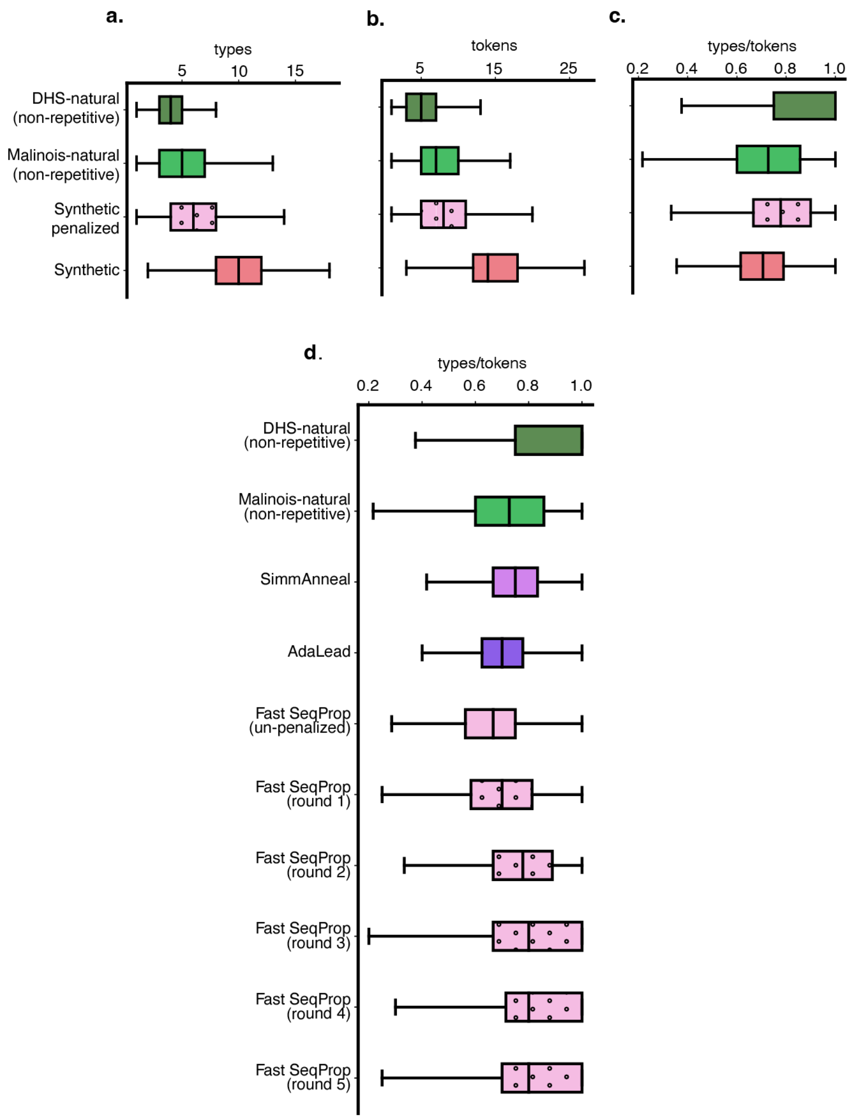

Supplementary Note 2 - Figure 4. Lexical analysis of motif content. (**a**) Individual synthetic sequences are composed of more unique enriched sequence motifs than natural sequences. Distribution of unique motifs (types) in each sequence, binned by CRE proposal method. Boxes demarcate the 25th, 50th, and 75th percentile values, while whiskers indicate the outermost point within 1.5 times the interquartile range from the edges of the boxes. Top-to-bottom: n=12,000; 12,000; 15,000; 36,000. (**b**) Synthetic sequences contain more instances of enriched motifs than natural sequences. Distribution of total motif instances (tokens) in each sequence, binned by CRE proposal method. Boxes demarcate the 25th, 50th, and 75th percentile values, while whiskers indicate the outermost point within 1.5 times the interquartile range from the edges of the boxes. n sizes as in **a**. (**c**) Distribution of type:token in each sequence, binned by CRE proposal method. Boxes demarcate the 25th, 50th, and 75th percentile values, while whiskers indicate the outermost point within 1.5 times the interquartile range from the edges of the boxes. n sizes as in **a**. (**d**) Motif penalization reduces motif redundancy in synthetic CREs. Boxplots are similar to c. except synthetic elements are broken up into more granular bins. Boxes demarcate the 25th, 50th, and 75th percentile values, while whiskers indicate the outermost point within 1.5 times the interquartile range from the edges of the boxes. Top-to-bottom: n=12,000; 12,000; 12,000; 12,000; 12,000; 3,000; 3,000; 3,000; 3,000; 3,000.

Supplementary Note 3: Experimental reproducibility of MPRA across projects

A set of 594 control elements shared with the training data libraries confirms the high reproducibility of MPRA measurements across experiments (Pearson's r 0.97, 0.81, and 0.98 for K562, HepG2, and SK-N-SH, respectively; **Supplementary Note 3 - Figure 1**).

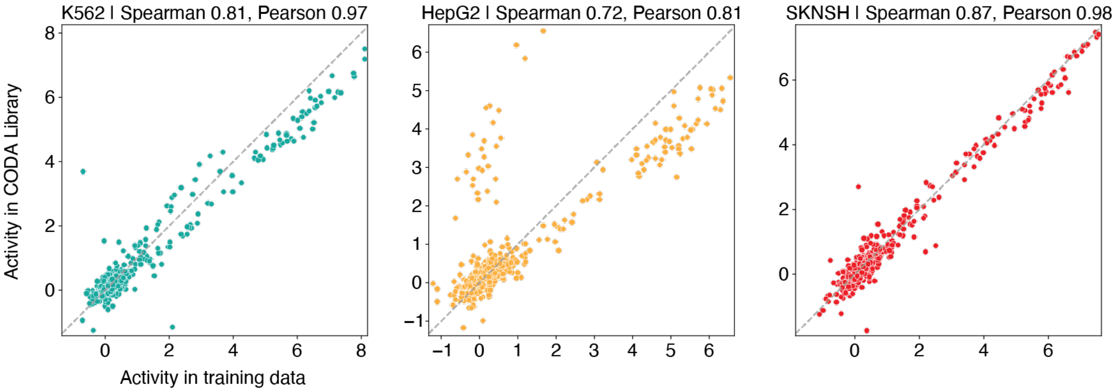

Supplementary Note 3 - Figure 1. MPRA measurements for individual elements are reproducible between different experiments and libraries. MPRA activity measurements made in the training data plotted on the x-axis are highly correlated with later measurements made in the CODA library on the y-axis. Measurements for n=592 elements were made in K562 (teal), HepG2 (gold), and SK-N-SH (red).

Supplementary Note 4: Validation of contribution scores for model interpretation

In order to validate that our contribution score method accurately reflects how single nucleotides impact model predictions, we systematically disrupted sequence segment blocks of positive, negative, and neutral contributions (**Methods**). We consistently observed that disrupting blocks of positive contribution led to a decrease in predicted activity, while disrupting blocks of negative contribution resulted in an increase (**Supplementary Note 4 - Figure 1, Methods**). This alignment with expected effects supports the suitability of the contribution score method to interpret model predictions.

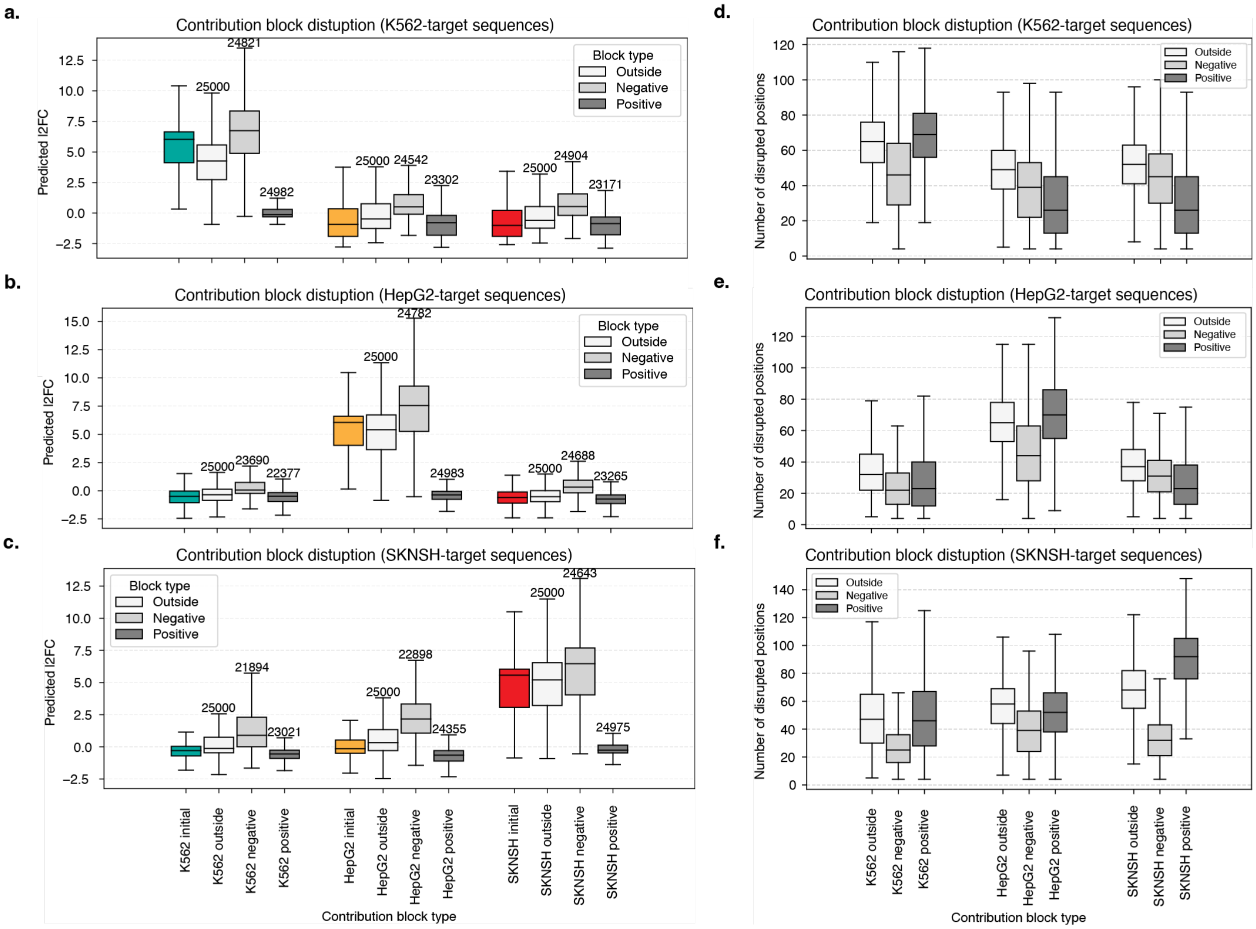

Supplementary Note 4 - Figure 1. Contribution block ablation. (**a**) Initial predicted activity in K562 (teal), HepG2 (gold), and SK-N-SH (red) of sequences targeting K562, n=25,000, x-axis labels shared with (c). Activity predictions of sequences with disrupted segments in negative (gray), positive (dark gray) contribution blocks, or outside blocks (light gray) in each cell type. The number above each gray box denotes the number of disrupted sequences. Left to right: n=25,000; 25,000; 24,821; 24,982; 25,000; 25,000; 24,542; 23,302; 25,000; 25,000; 24,904; 23,171. (**b**) Same as (a) but sequences targeting HepG2. Left to right: n=25,000; 25,000; 23,690; 22,377; 25,000; 25,000; 24,782; 24,983; 25,000; 25,000; 24,688; 23,265. (**c**) Same as (a) but sequences targeting SK-N-SH. Left to right: n=25,000; 25,000; 21,894; 23,021; 25,000; 25,000; 22,898; 24,355; 25,000; 25,000; 24,643; 24,975. (**d**) Number of positions disrupted in (a) in negative (gray), positive (dark gray) contribution blocks, or outside blocks (light gray) in each cell type. Left to right: n=25,000; 24,821; 24,982; 25,000; 24,542; 23,302; 25,000; 24,904; 23,171. (**e**) Same as (d) but disrupted in (b). Left to right: n=25,000; 23,690; 22,377; 25,000; 24,782; 24,983; 25,000; 24,688; 23,265. (**f**) Same as (d) but disrupted in (c). Left to right: n=25,000; 21,894; 23,021; 25,000; 22,898; 24,355; 25,000; 24,643; 24,975. (a-f) Boxes demarcate the 25th, 50th, 75th percentile values. Whiskers indicate the outermost point within 1.5 times the interquartile range from the box edges.

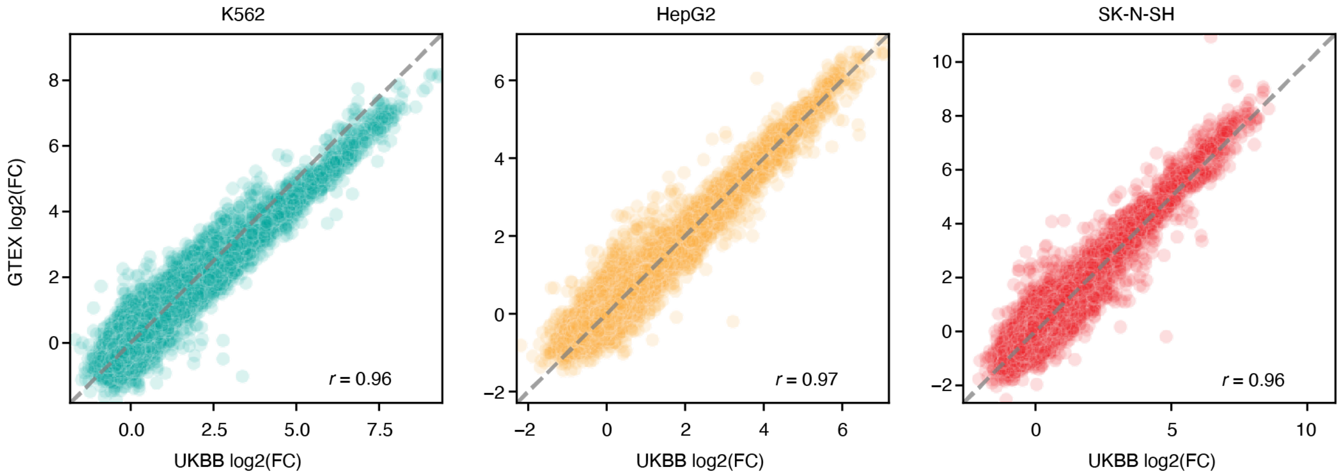

Supplementary Figure 1. MPRA library reproducibility. Scatter plots compare the $\log_2$(Fold-Change) ($\log_2$(FC)) of n=20,303 sequences shared between the UKBB and GTEx MPRA libraries, two libraries experimentally conducted independently from each other at distinct points of time. The *x*-axis corresponds to the $\log_2$(FC) as measured in UKBB, and the *y*-axis corresponds to the $\log_2$(FC) as measured in GTEx. The Pearson's correlation coefficient is shown in the right bottom corner. Oligos with a replicate $\log_2$(FC) standard error greater than 1 were omitted from the comparisons.

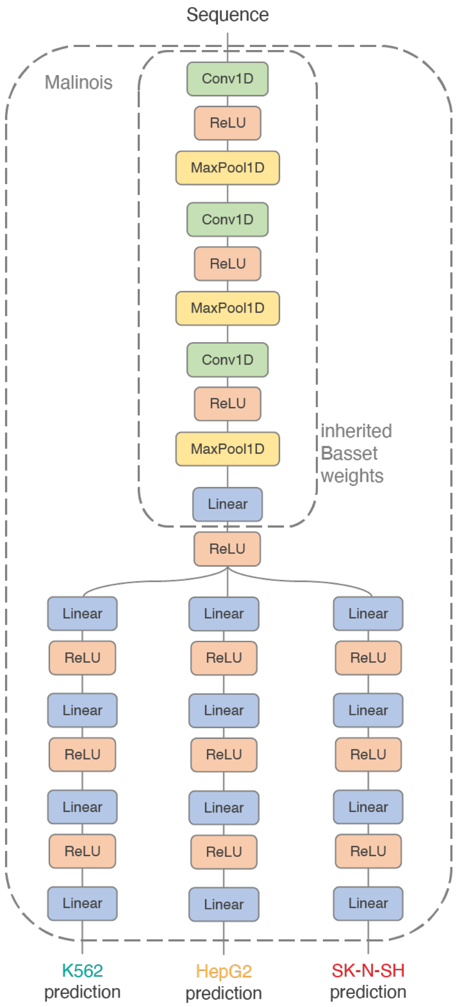

Supplementary Figure 2. Model schematic. Schematic of the Malinois model architecture. Malinois is composed of 3 convolutional layers, 1 shared linear layer, and 3 independent branches of 4 linear layers—1 branch for activity predictions in each cell type. All hidden layers are followed by rectified linear units while convolutional layers are also separated by pooling operations. Layers with weights inherited from Basset at the initiation of training are indicated.

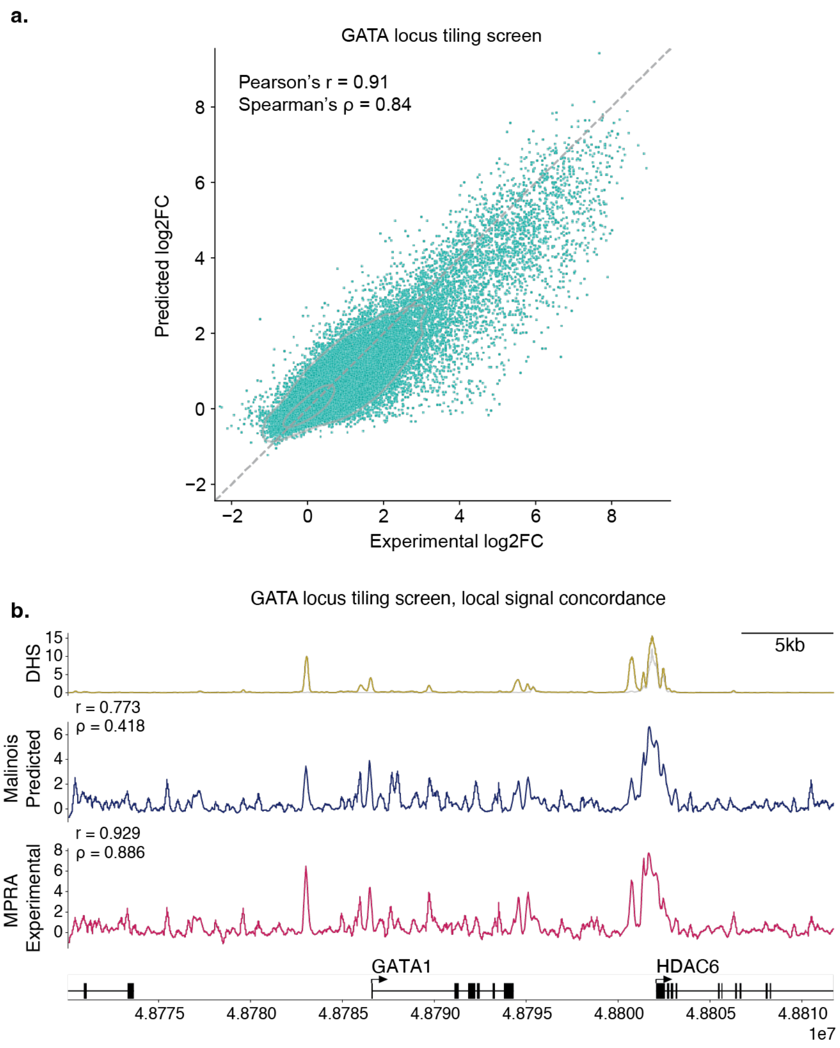

Supplementary Figure 3. Correlation of Malinois predictions and empirical MPRA tiling data. (**a**) Malinois predictions are highly correlated with empirical MPRA measurements of tiled sequences in the GATA locus (chrX:47,785,602:49,880,397) in K562 (Pearson's *r* = 0.91, Spearman's ρ = 0.84). *X*-axis and *y*-axis correspond to empirical measurements and Malinois predictions, respectively for oligos in the library (n = 51242 oligos). Sequences which overlap with oligos from the validation data split used for model selection were removed from this plot and correlation calculations (n = 2420 oligos omitted). Additionally, oligos with a replicate log_2FC standard error greater than 1 in any cell type were omitted from the plots. (**b**) Malinois predictions projected onto the genome are correlated with empirical MPRA projections and DHS signal in regions with active CREs. Pearson's *r* and Spearman's rho are calculated for the predicted track compared to either DHS (upper) or MPRA (lower).

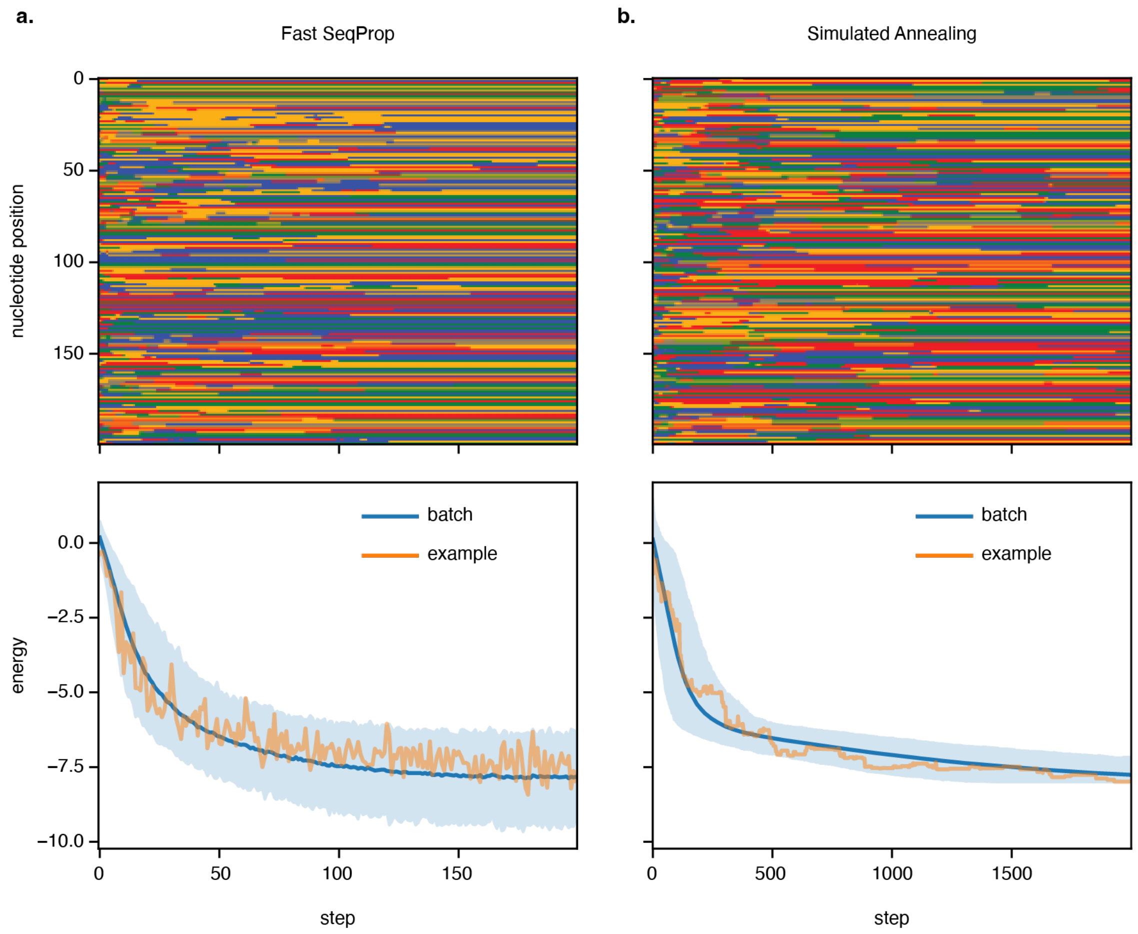

Supplementary Figure 4. Example sequence generation trajectory (**a**) Fast SeqProp can generate sequences that are predicted to minimize an objective function. A trajectory was generated for 512 sequences using 200 update steps. Top: An example trajectory of a single sequence in the trajectory. Color represents nucleotide identity along the sequence after each update during the algorithm (A: Green, C: Blue, G: Yellow, T: Red). Bottom: The predicted objective value of sequences at each step of Fast SeqProp. The mean is indicated by the line and bounds of the 95-percentile data range are shaded light blue. The example displayed above is indicated by the orange line. (**b**) Same as **a** but generated using 2000 steps of simulated annealing.

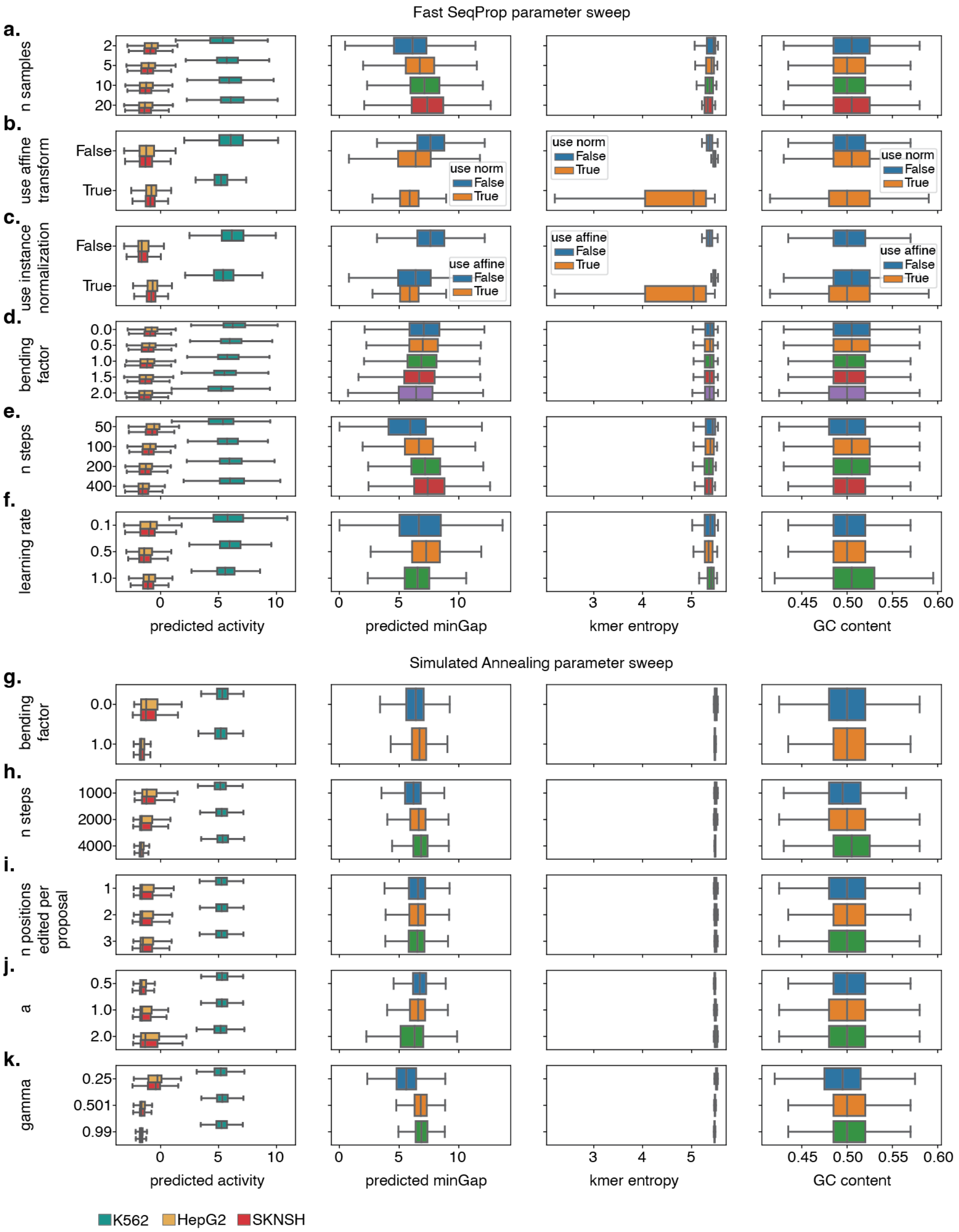

Supplementary Figure 5. Screening sequence design hyperparameters for generating synthetic CREs. Different hyperparameter combinations for Fast SeqProp (a)-(f) and Simulated Annealing (g)-(k) were tested to generate predicted K562-specific synthetic CREs. Predicted log2-fold-change, predicted minGap activity, 4-mer heterogeneity, and GC content was measured for each sequence and plotted as a function of hyperparameter choices. (a)-(k) Boxes demarcate the 25th, 50th, and 75th percentile values, while whiskers indicate the outermost point within 1.5 times the interquartile range from the edges of the boxes. The following number of elements were generated for each hyperparameter setting plotted on the y-axis of subpanels: (a) n=240,000; 240,000; 240,000 (b) n=720,000; 240,000 (c) n=480,000; 480,000 (d) n=192,000; 192,000; 192,000; 192,000; 192,000 (e) n=240,000; 240,000; 240,000; 240,000 (f) n=320,000; 320,000; 320,000 (g) n=81,000; 81,000 (h)-(k) n=54,000; 54,000; 54,000.

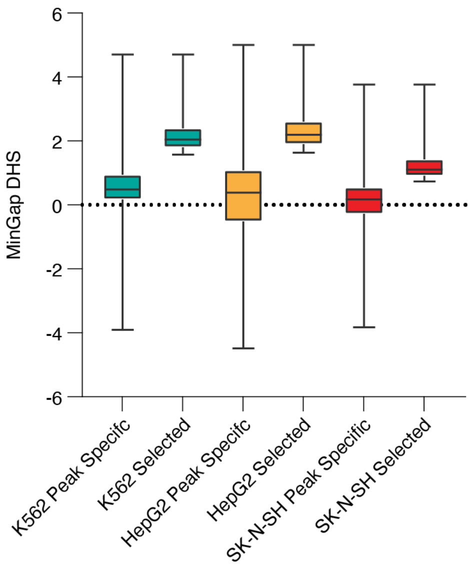

Supplementary Figure 6. DHS signal specificity of DHS-natural sequences. DHS specificity as measured by MinGap of log2 of DHS signal counts for specific peaks, and the selected 4,000 peaks for MPRA. Boxes demarcate the 25th, 50th, and 75th percentile values, while whiskers indicate maximum and minimum (left-to-right: n=35,894; 4,000; 27,310; 4,000; 20,773; 4,000).

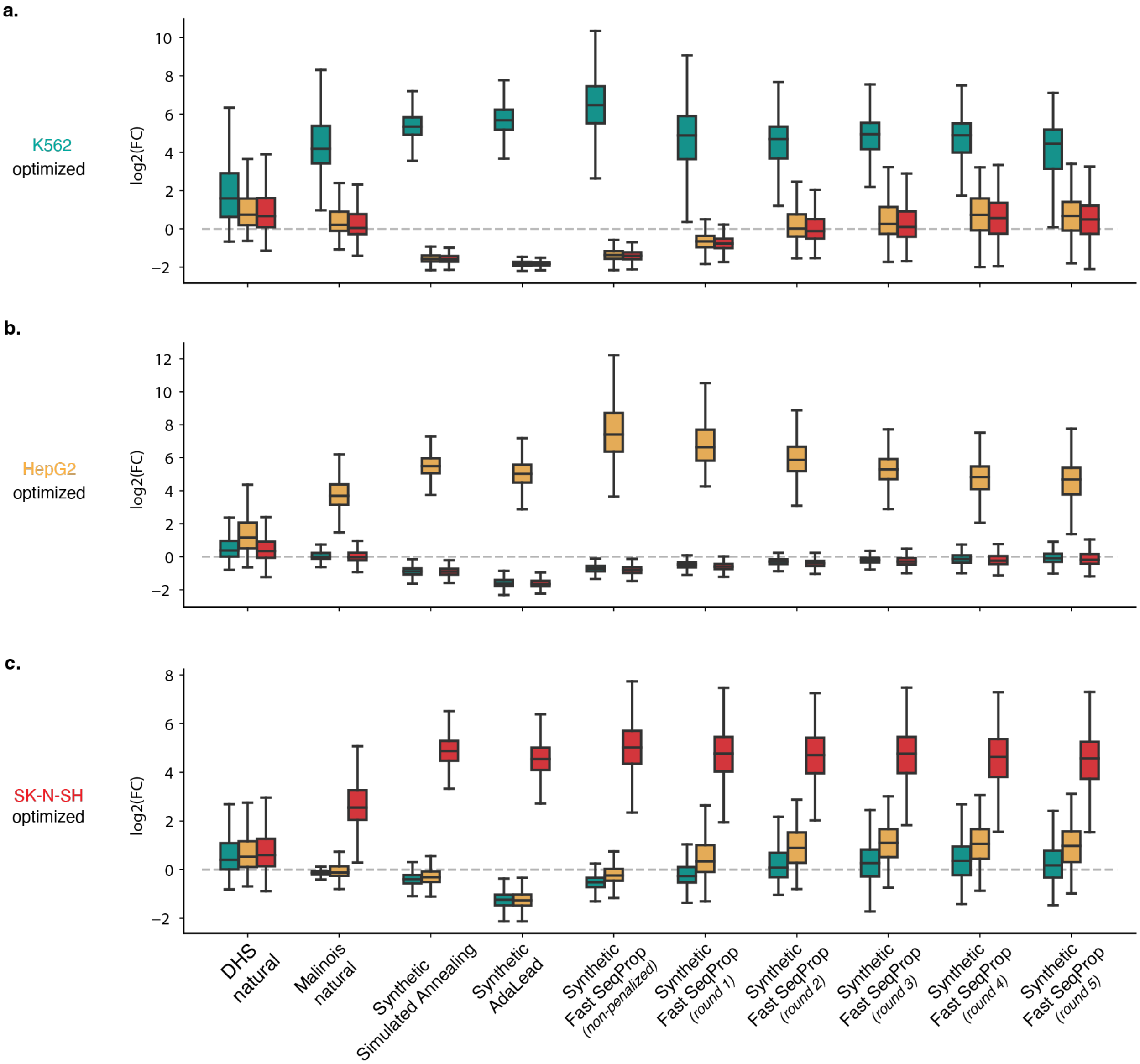

Supplementary Figure 7. Predicted library activity. (**a**) Distribution of projected activity in K562 (teal), HepG2 (gold), and SK-N-SH (red) for candidate CREs predicted to drive K562-specific transcription. Boxes demarcate the 25th, 50th, and 75th percentile values, while whiskers indicate the outermost point within 1.5 times the interquartile range from the edges of the boxes. Left-to-right: n=4,000; 4,000; 4,000; 4,000; 4,000; 1,000; 1,000; 1,000; 1,000; 1,000. (**b**) Same as **a,** but for candidate CREs predicted to drive HepG2-specific transcription. n sizes same as **a**. (**c**) Same as **a** and **b,** but for candidate CREs predicted to drive SK-N-SH-specific transcription. n sizes same as **a**.

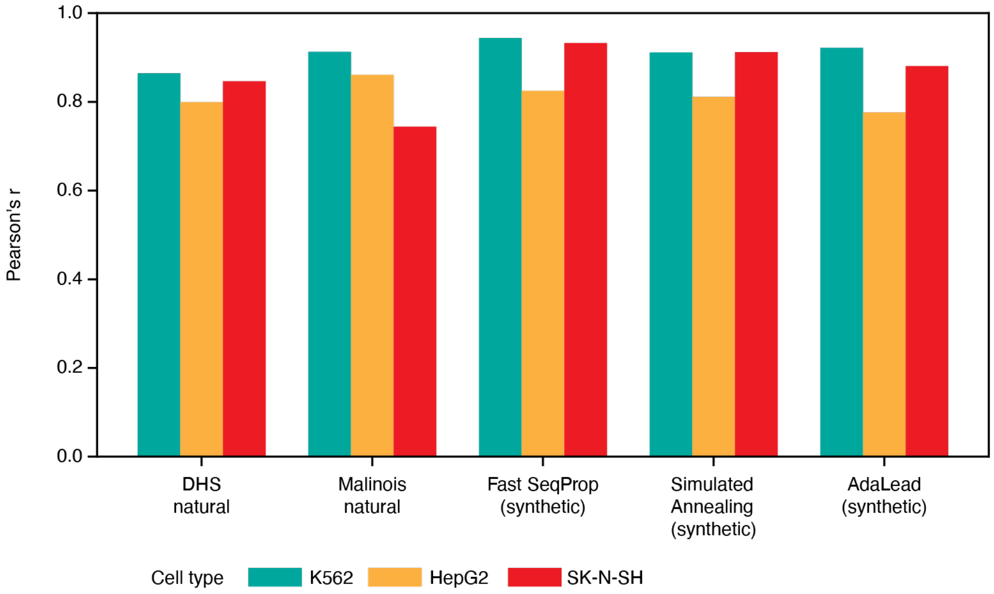

Supplementary Figure 8. Granular Malinois prediction performance of CODA library. Pearson correlation coefficient values between Malinois activity predictions and MPRA empirical measurements in K562 (teal), HepG2 (gold), and SK-N-SH (red) of the CODA library broken down by method group. Number of sequences n=10,747; 10,941; 11,336; 11,181; 10,944. All *p*-values < 1e-300.

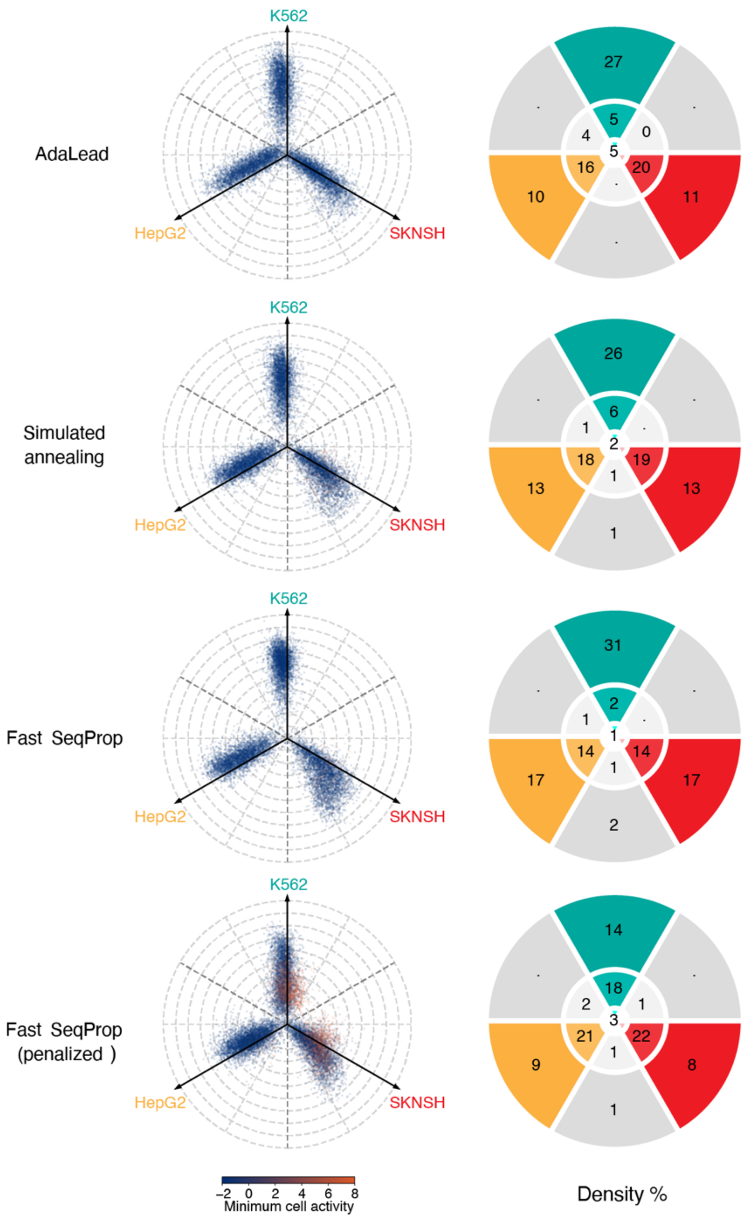

Supplementary Figure 9. Complete propeller plots. Propeller plots of refined synthetic subsets of the library (see **Figure 2e** legend for description of coordinate system). Number of sequences top to bottom n=10,944; 11,181; 11,336; 14,401.

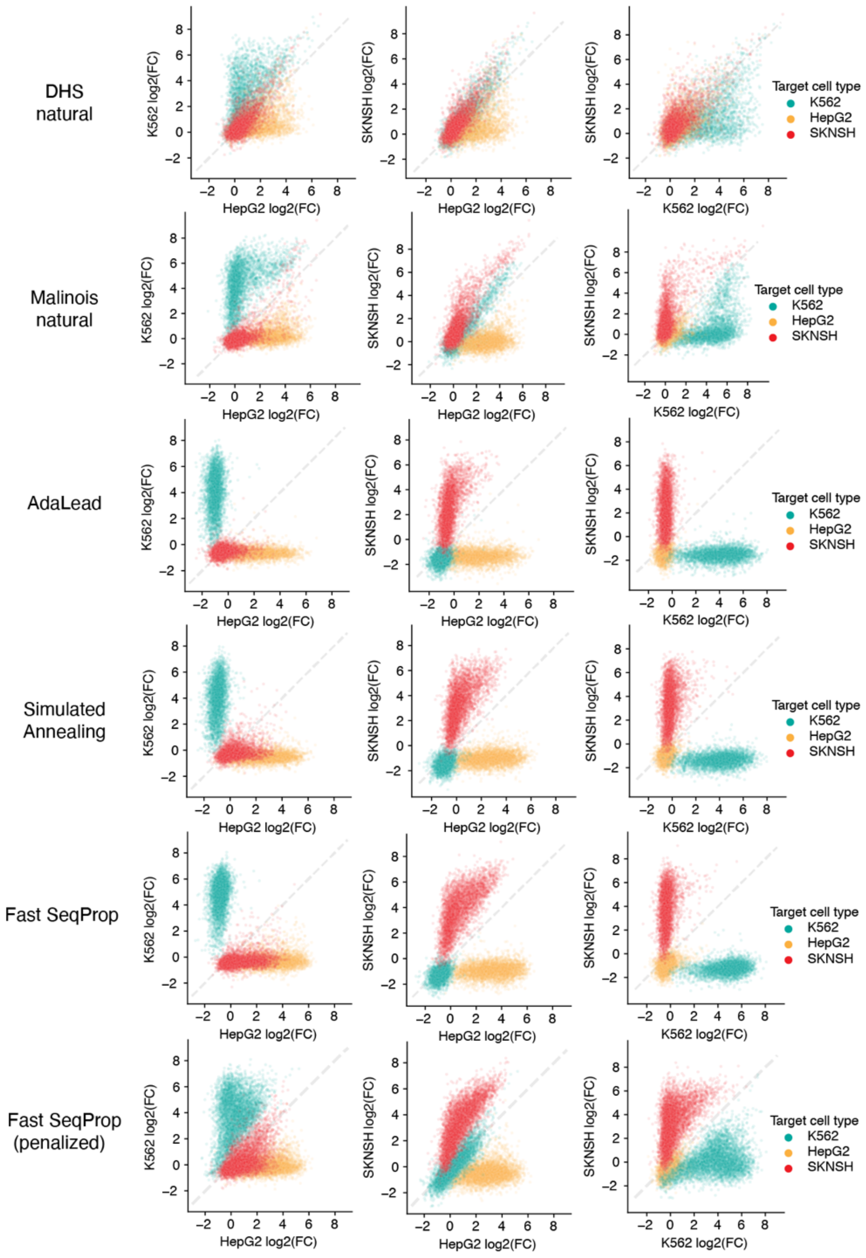

Supplementary Figure 10. Cell type activity comparisons. Scatter plots comparing empirical log2(Fold-Change) activity in each pair of cell types for each design group. Color indicates the target cell type for which sequences were designed (synthetic) or selected (natural). Number of sequences top to bottom n=10,747; 10,941; 10,944; 11,181; 11,336; 14,401.

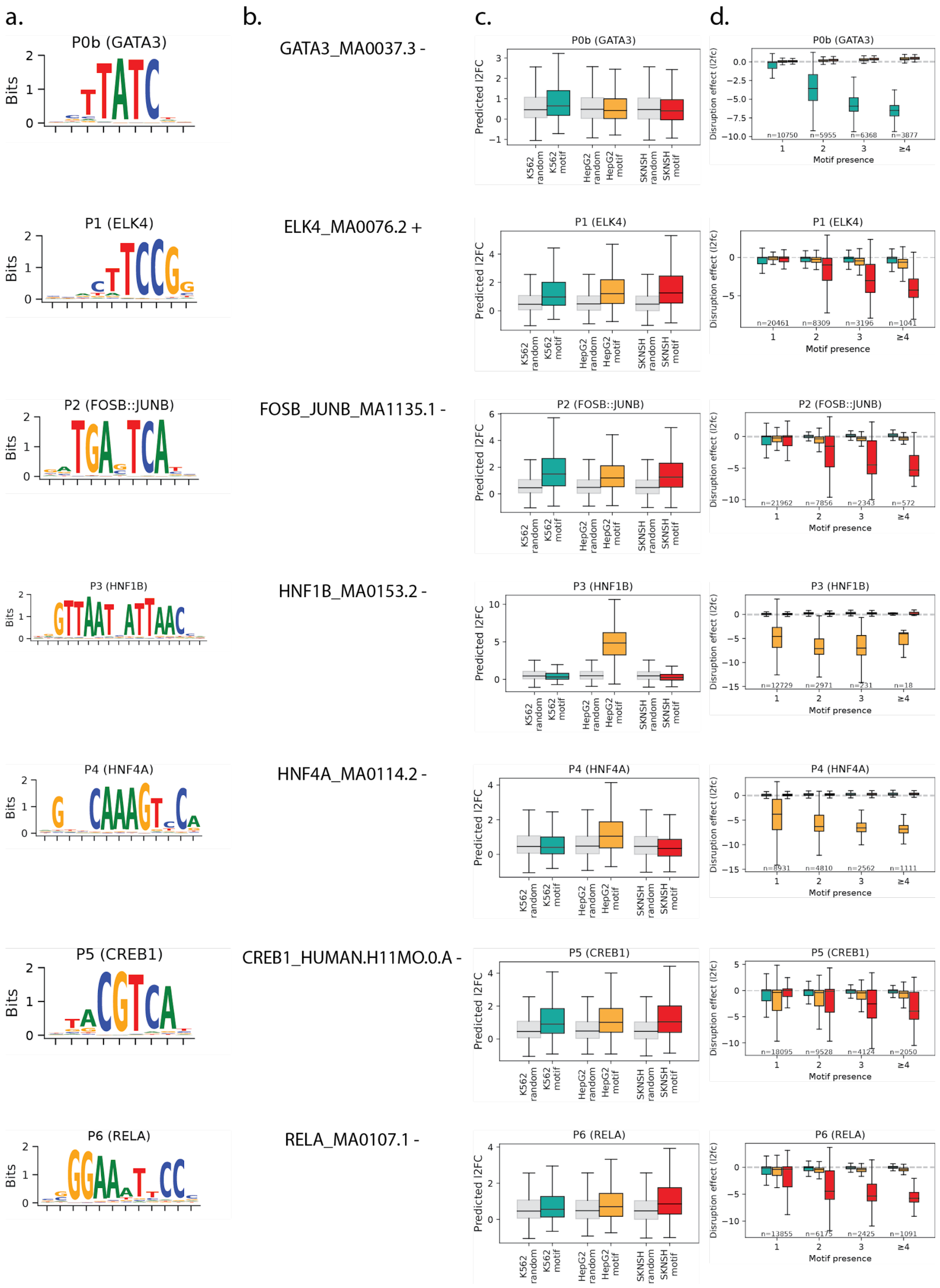
a.
b.
c.
d.
P0b (GATA3)
GATA3_MA0037.3 -
P1 (ELK4)
ELK4_MA0076.2 +
P2 (FOSB::JUNB)
FOSB_JUNB_MA1135.1 -
P3 (HNF1B)
HNF1B_MA0153.2 -
P4 (HNF4A)
HNF4A_MA0114.2 -
P5 (CREB1)
CREB1_HUMAN.H11MO.0.A -
P6 (RELA)
RELA_MA0107.1 -
Bits
Predicted l2FC
K562 random
K562 motif
HepG2 random
HepG2 motif
SKNSH random
SKNSH motif
Disruption effect (l2fc)
Motif presence
1
2
3
≥4
n=10750
n=5955
n=6368
n=3877
n=20461
n=8309
n=3196
n=1041
n=21962
n=7856
n=2343
n=572
n=12729
n=2971
n=231
n=18
n=8931
n=4810
n=2562
n=1111
n=18095
n=9528
n=4124
n=2050
n=13855
n=6175
n=2425
n=1091

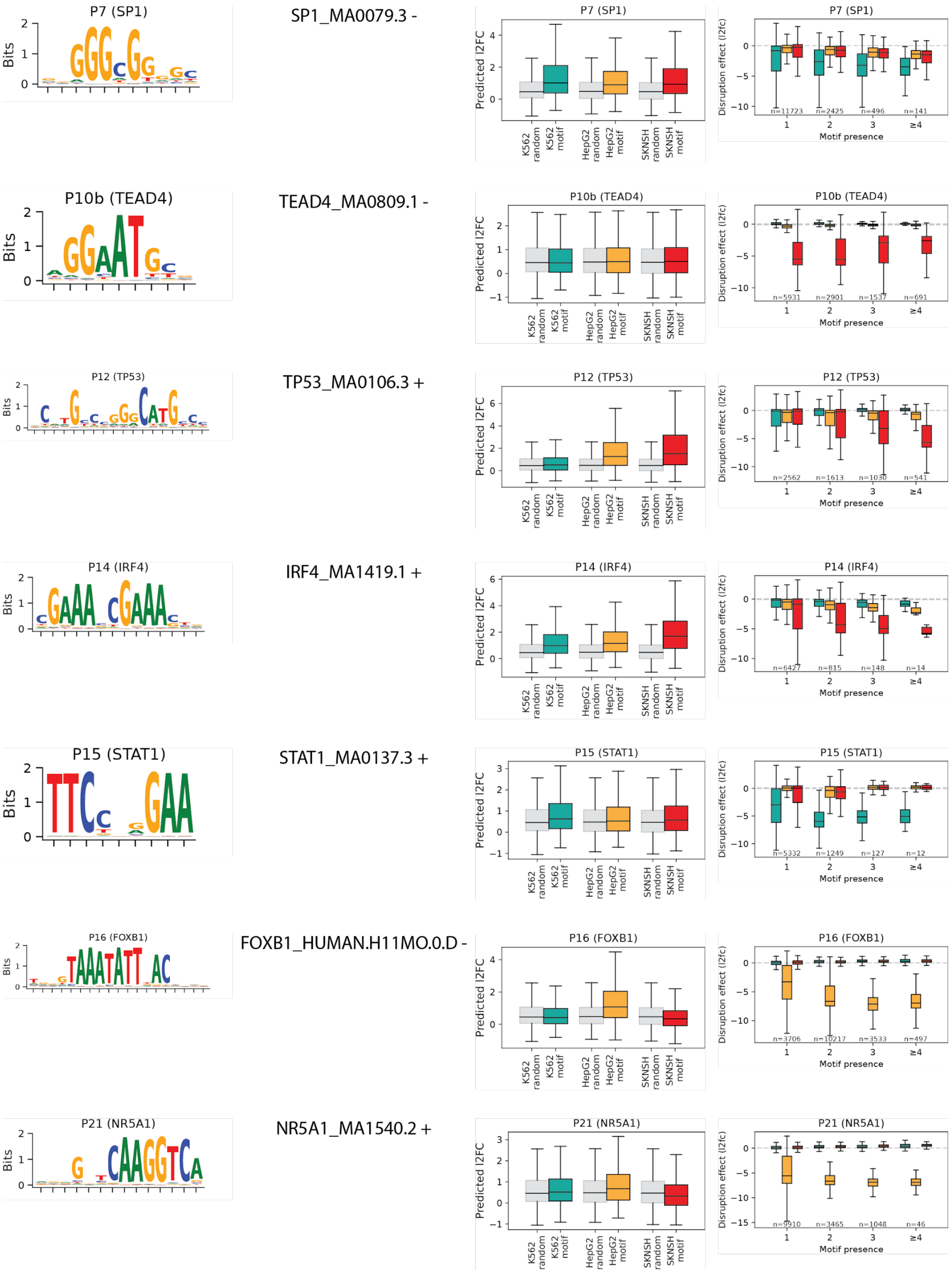
P7 (SP1)
SP1_MA0079.3 -
P10b (TEAD4)
TEAD4_MA0809.1 -
P12 (TP53)
TP53_MA0106.3 +
P14 (IRF4)
IRF4_MA1419.1 +
P15 (STAT1)
STAT1_MA0137.3 +
P16 (FOXB1)
FOXB1_HUMAN.H11MO.0.D -
P21 (NR5A1)
NR5A1_MA1540.2 +
Bits
Predicted l2FC
K562 random
K562 motif
HepG2 random
HepG2 motif
SKNSH random
SKNSH motif
Disruption effect (l2fc)
Motif presence
n=11723
n=2425
n=496
n=141
n=5931
n=2901
n=1537
n=691
n=2562
n=1613
n=1030
n=541
n=6427
n=815
n=148
n=14
n=5332
n=1249
n=127
n=12
n=3706
n=10217
n=3533
n=497
n=9910
n=3465
n=1048
n=46

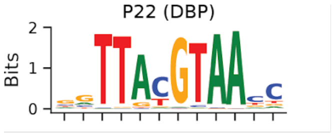

DBP_MA0639.1 -

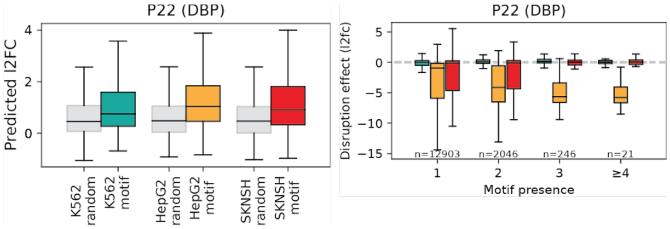

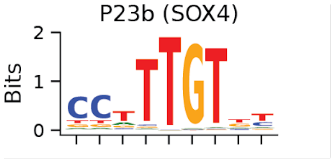

SOX4_MA0867.2 -

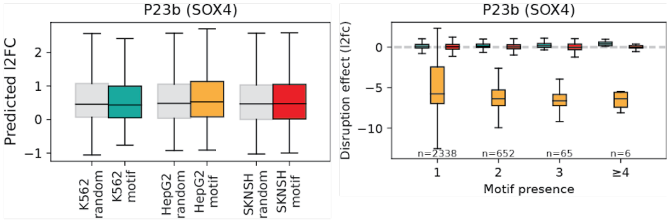

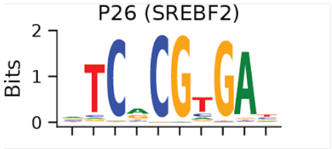

SREBF2_MA0828.1 +

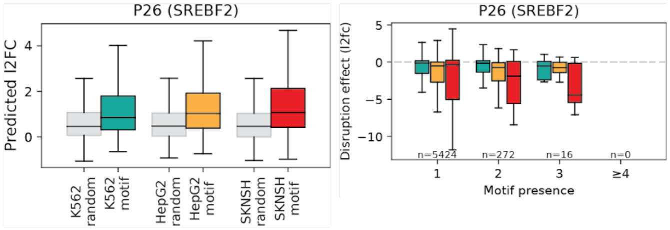

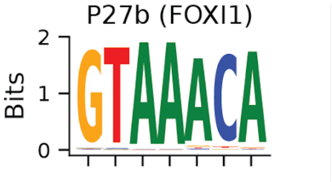

FOXI1_MA0042.2 +

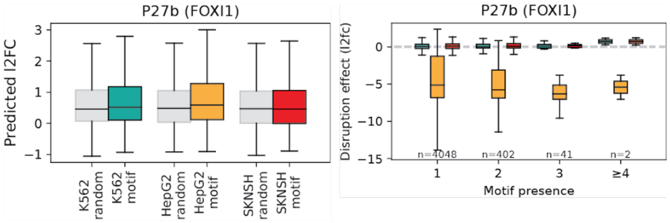

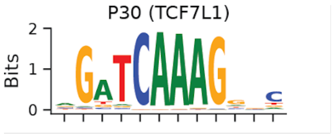

TCF7L1_MA1421.1 +

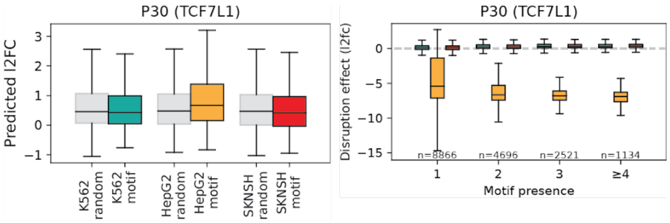

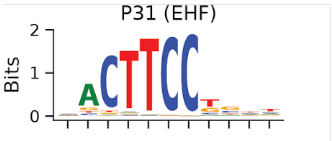

EHF_MA0598.3 +

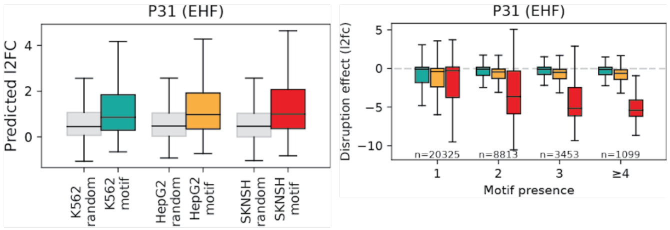

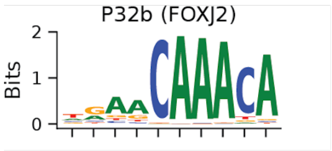

FOXJ2_HUMAN.H11MO.0.C -

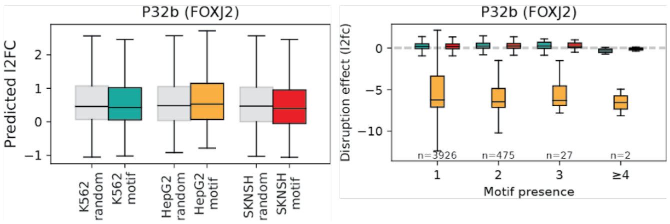

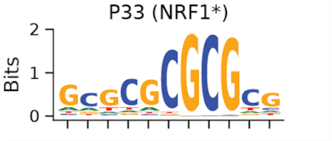

*NRF1_MA0506.1 +

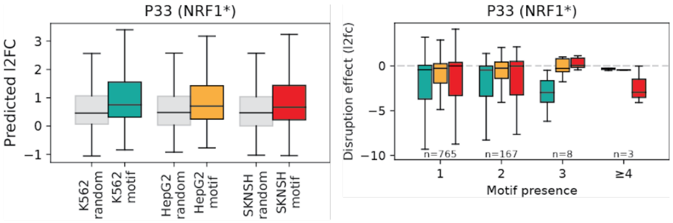

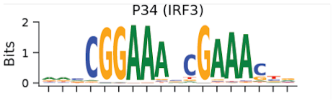

IRF3_MA1418.1 +

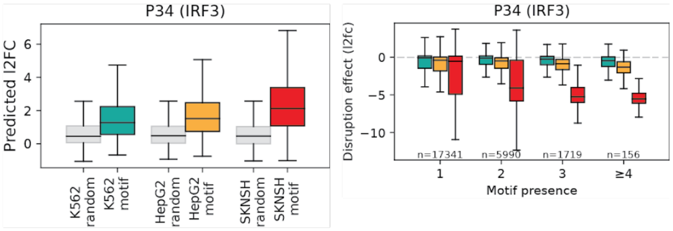

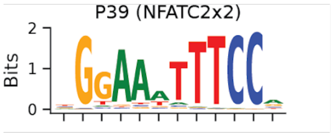

NFATC2_MA0152.1 -::+

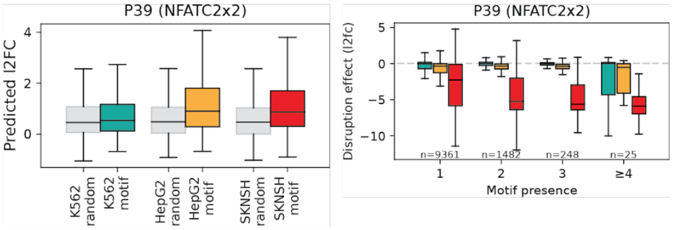

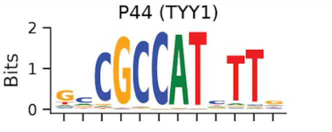

TYY1_HUMAN.H11MO.0.A -

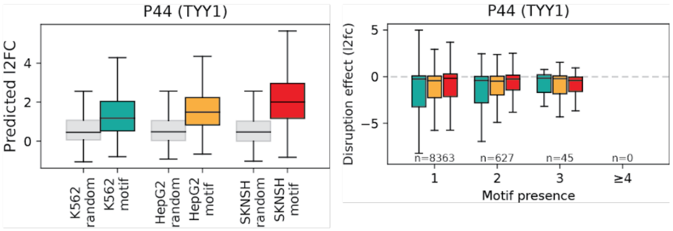

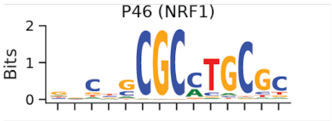

NRF1_MA0506.1 +

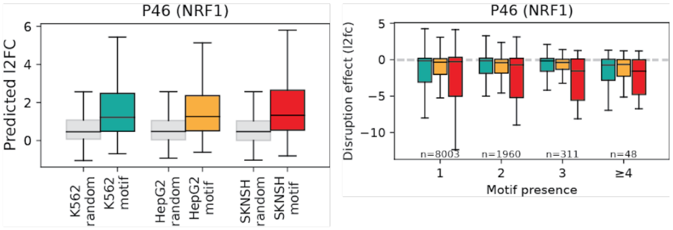

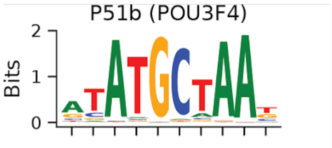

POU3F4_MA0789.1 +

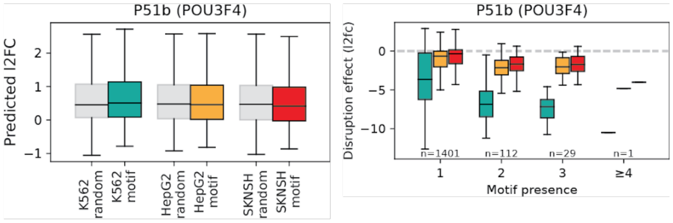

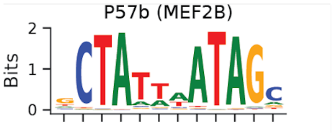

MEF2B_MA0660.1 -

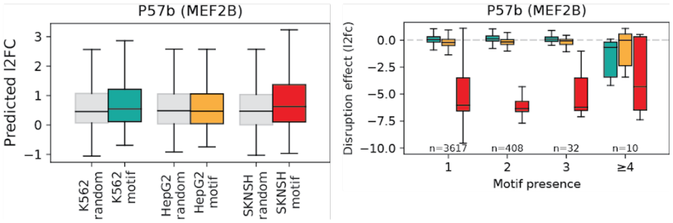

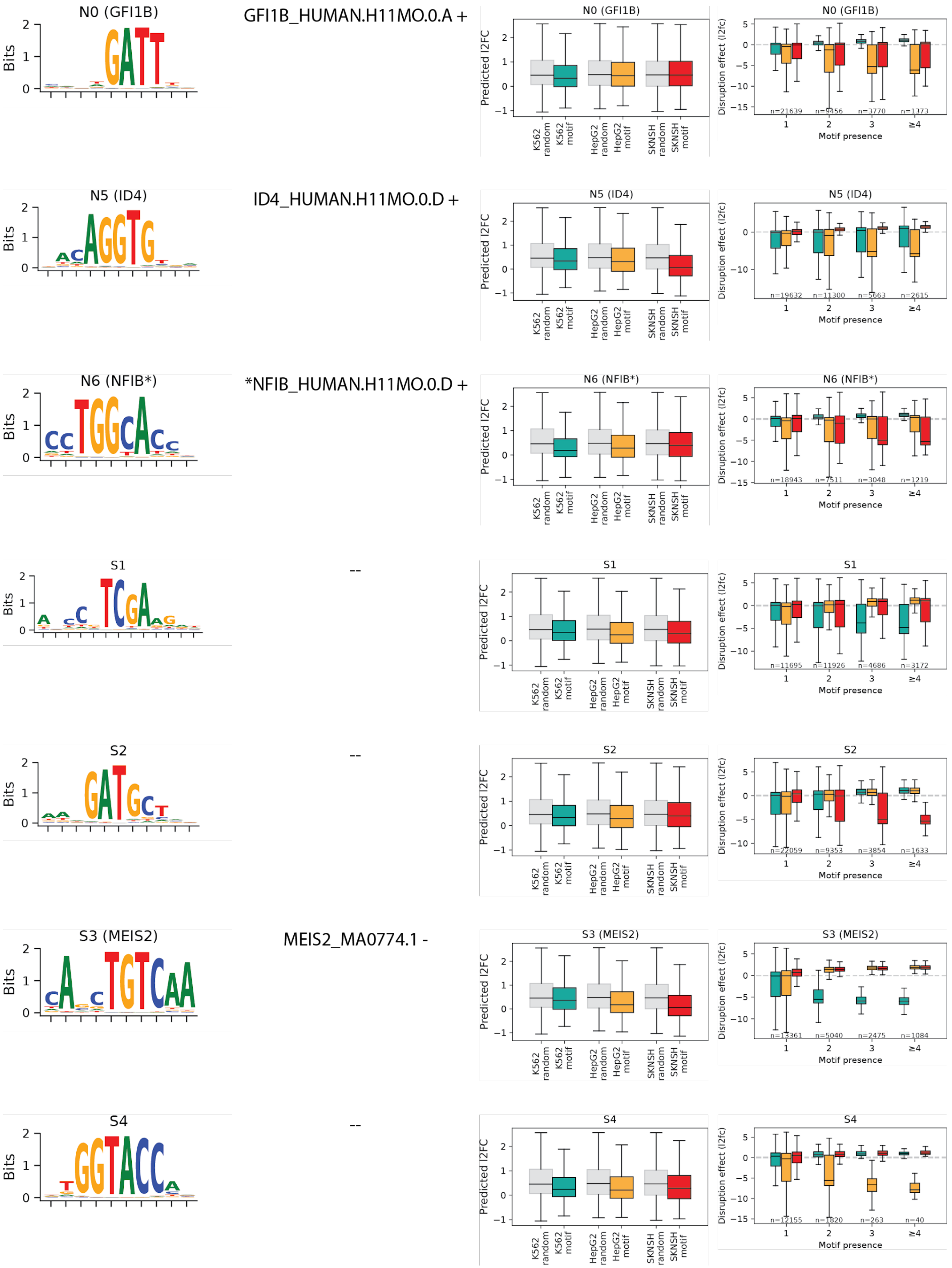
N0 (GFI1B)
GFI1B_HUMAN.H11MO.0.A +
N5 (ID4)
ID4_HUMAN.H11MO.0.D +
N6 (NFIB*)
*NFIB_HUMAN.H11MO.0.D +
S1
--
S2
--
S3 (MEIS2)
MEIS2_MA0774.1 -
S4
--
Bits
Predicted l2FC
K562 random
K562 motif
HepG2 random
HepG2 motif
SKNSH random
SKNSH motif
Disruption effect (l2fc)
Motif presence
1
2
3
≥4
n=21639
n=9456
n=3770
n=1373
n=19632
n=11300
n=5663
n=2615
n=18943
n=7511
n=3048
n=1219
n=11695
n=11926
n=4686
n=3172
n=22059
n=9353
n=3854
n=1633
n=13361
n=5040
n=2475
n=1084
n=12155
n=1820
n=263
n=40

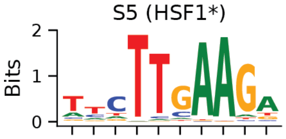

*HSF1_HUMAN.H11MO.1.A +

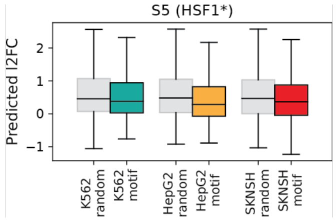

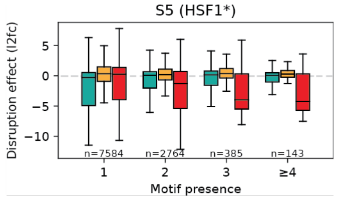

Supplementary Figure 11. Predicted functionality of core motifs. (**a**) Information-Content logos of core motifs. The *x*-axis and *y*-axis denote positions and bits, respectively. (**b**) Matches to known human TF binding motifs in JASPAR or HOCOMOCO. An asterisk at the beginning of the name indicates a moderate match with 1 < *E*-value < 10. No name (dashes) indicates that any possible match had an *E*-value < 10. Otherwise, the name corresponds to a match with an *E*-value < 1. The symbols +/- at the end of the name indicate the orientation of the match as forward or reverse complement respectively. (**c**) Activity predictions of sequences consisting of randomly sampled motif instances in the center and randomly background-sampled flanks in K562 (teal), HepG2 (gold), and SK-N-SH (red), along with activity predictions of fully random background-sampled sequences in K562, HepG2, and SK-N-SH (all in light gray). Boxes demarcate the 25th, 50th, and 75th percentile values, while whiskers indicate the outermost point within 1.5 times the interquartile range from the edges of the boxes. Number of sequences: n=5,000 for all boxes. (**d**) Predicted activity effect of disrupting all motif instances in the sequence library binned my motif presence score. Teal, gold, and red boxes correspond to effects to the predicted activity in K562, HepG2, and SK-N-SH, respectively. The *y*-axis corresponds to the activity prediction of the original (undisrupted) sequences minus the activity prediction of sequences with disrupted motif instances replaced by randomly background-sampled segments. The integer *n* below each bin of boxes indicates the number of sequences present in each motif score bin. Boxes demarcate the 25th, 50th, and 75th percentile values, while whiskers indicate the outermost point within 1.5 times the interquartile range from the edges of the boxes. Number of sequences is denoted below each set of boxes.

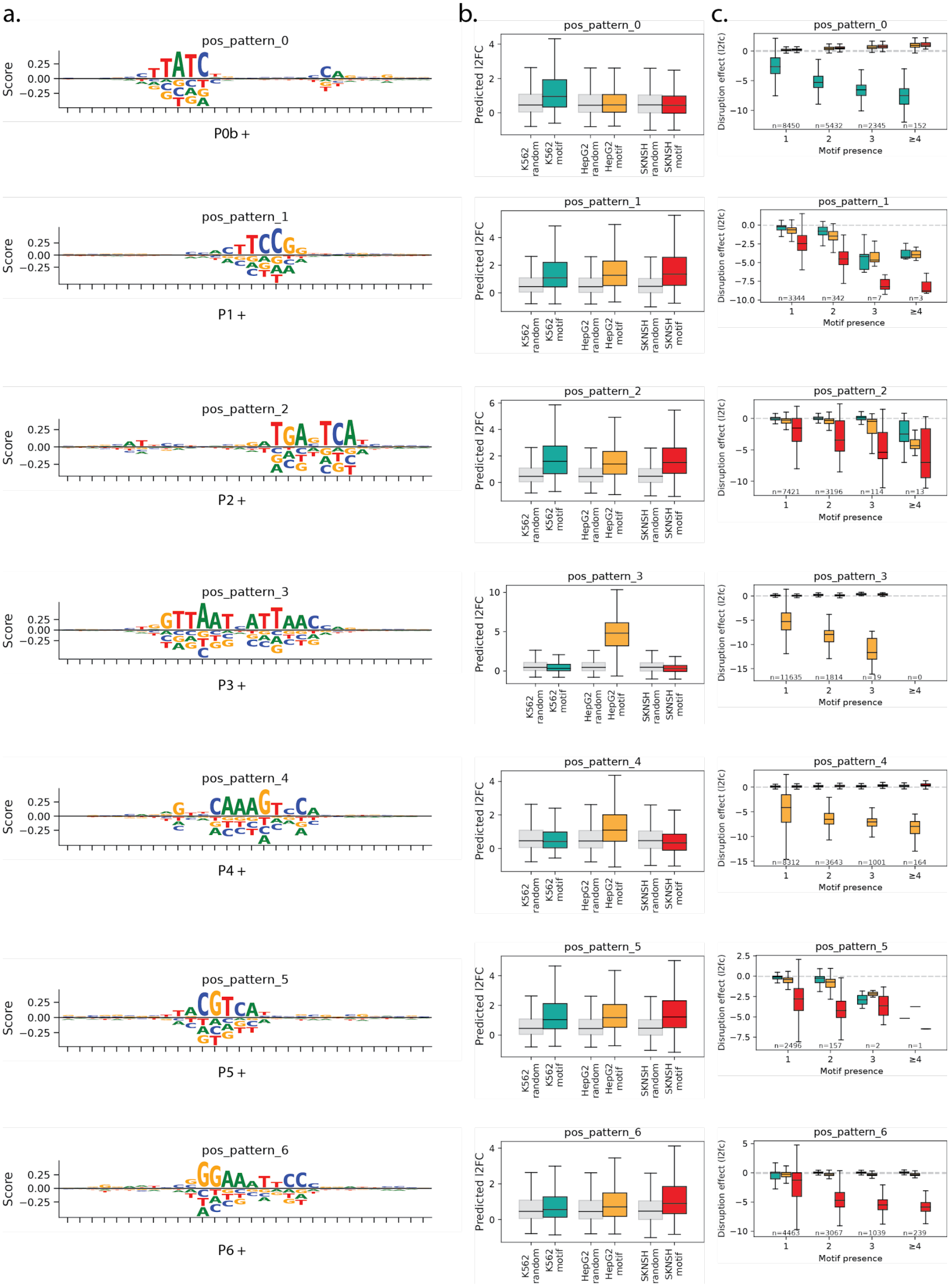
a.
b.
c.
pos_pattern_0
Score
0.25
0.00
−0.25
P0b +
Predicted l2FC
K562 random
K562 motif
HepG2 random
HepG2 motif
SKNSH random
SKNSH motif
Disruption effect (l2fc)
n=8450
n=5432
n=2345
n=152
Motif presence
1
2
3
≥4
pos_pattern_1
P1 +
n=3344
n=342
n=7
n=3
pos_pattern_2
P2 +
n=7421
n=3196
n=114
n=13
pos_pattern_3
P3 +
n=11635
n=1814
n=19
n=0
pos_pattern_4
P4 +
n=8312
n=3643
n=1001
n=164
pos_pattern_5
P5 +
n=2496
n=157
n=2
n=1
pos_pattern_6
P6 +
n=4463
n=3067
n=1039
n=239

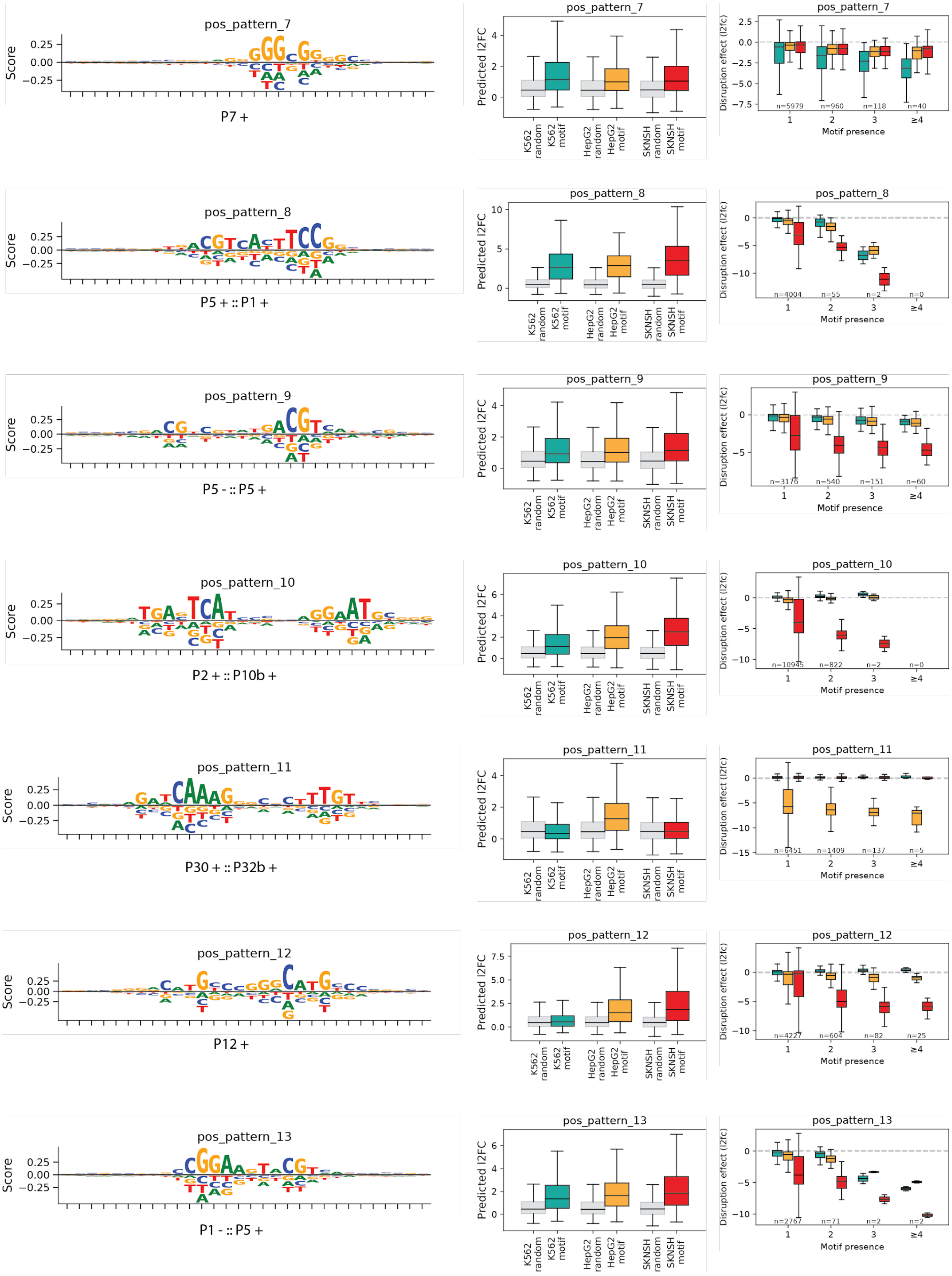

pos_pattern_7
Score
P7 +
Predicted l2FC
K562 random
K562 motif
HepG2 random
HepG2 motif
SKNSH random
SKNSH motif
Disruption effect (l2fc)
Motif presence
n=5979
n=960
n=118
n=40
pos_pattern_8
P5 + :: P1 +
n=4004
n=55
n=2
n=0
pos_pattern_9
P5 - :: P5 +
n=3176
n=540
n=151
n=60
pos_pattern_10
P2 + :: P10b +
n=10945
n=822
n=2
n=0
pos_pattern_11
P30 + :: P32b +
n=6451
n=1409
n=137
n=5
pos_pattern_12
P12 +
n=4227
n=604
n=82
n=25
pos_pattern_13
P1 - :: P5 +
n=2767
n=71
n=2
n=2

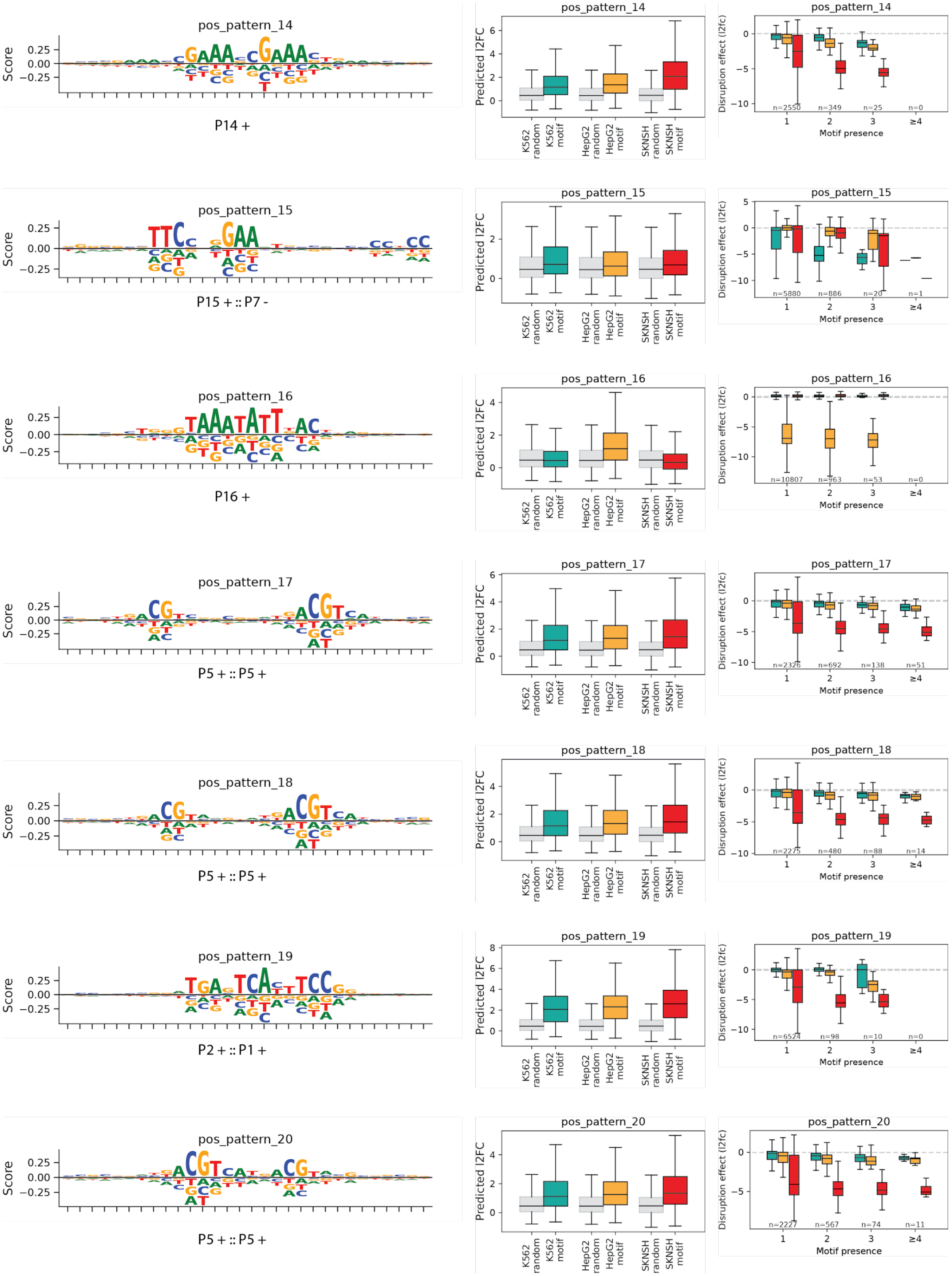

pos_pattern_14
Score
P14 +
Predicted l2FC
K562 random
K562 motif
HepG2 random
HepG2 motif
SKNSH random
SKNSH motif
Disruption effect (l2fc)
n=2550
n=349
n=25
n=0
Motif presence
pos_pattern_15
P15 + :: P7 -
n=5880
n=886
n=20
n=1
pos_pattern_16
P16 +
n=10807
n=963
n=53
n=0
pos_pattern_17
P5 + :: P5 +
n=2326
n=692
n=138
n=51
pos_pattern_18
P5 + :: P5 +
n=2275
n=480
n=88
n=14
pos_pattern_19
P2 + :: P1 +
n=6524
n=98
n=10
n=0
pos_pattern_20
P5 + :: P5 +
n=2227
n=567
n=74
n=11

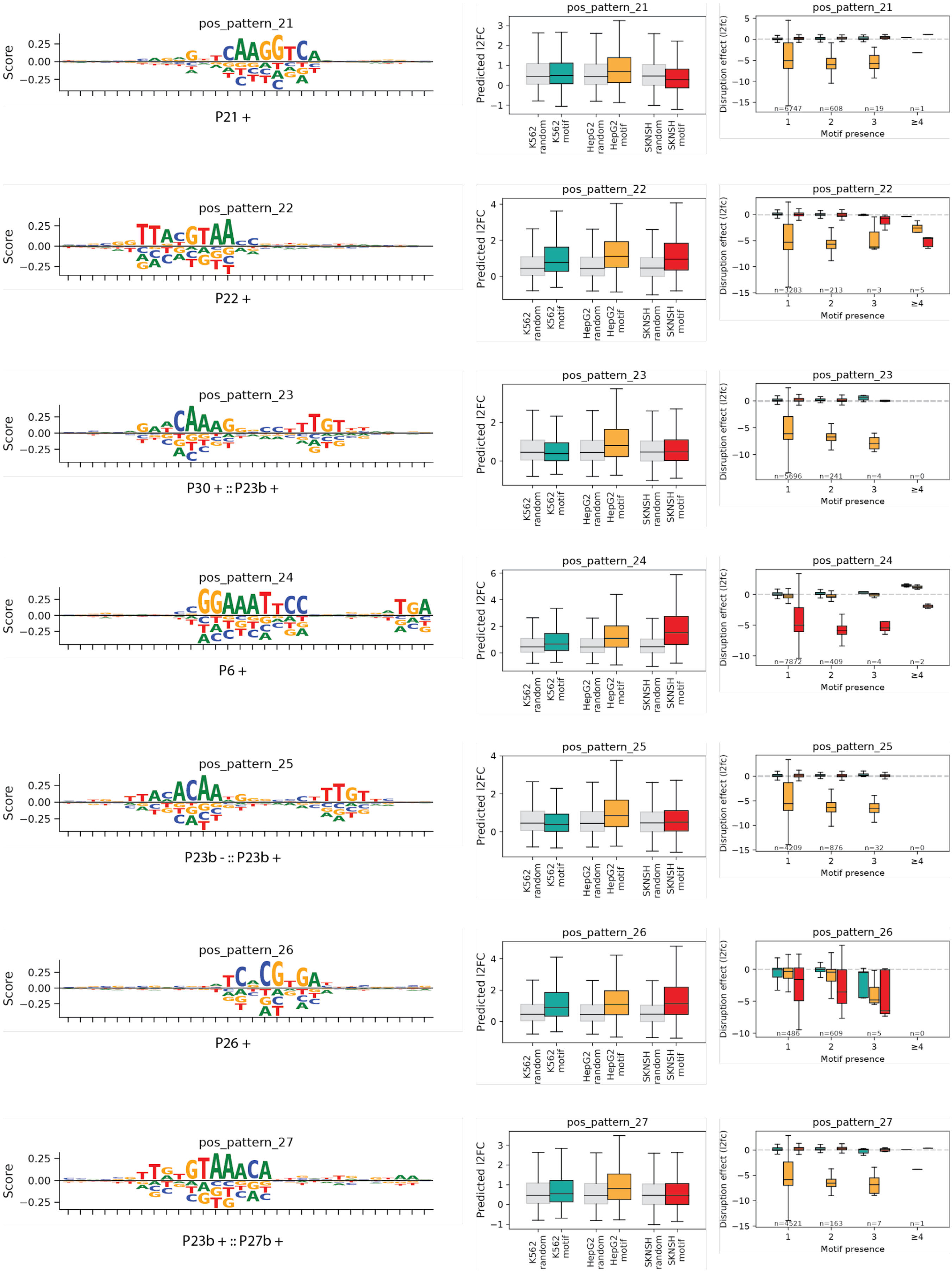
pos_pattern_21
Score
P21 +
Predicted l2FC
K562 random
K562 motif
HepG2 random
HepG2 motif
SKNSH random
SKNSH motif
Disruption effect (l2fc)
n=6747
n=608
n=19
n=1
Motif presence
pos_pattern_22
P22 +
n=3283
n=213
n=3
n=5
pos_pattern_23
P30 + :: P23b +
n=5696
n=241
n=4
n=0
pos_pattern_24
P6 +
n=7872
n=409
n=4
n=2
pos_pattern_25
P23b - :: P23b +
n=4209
n=876
n=32
n=0
pos_pattern_26
P26 +
n=486
n=609
n=5
n=0
pos_pattern_27
P23b + :: P27b +
n=4521
n=163
n=7
n=1

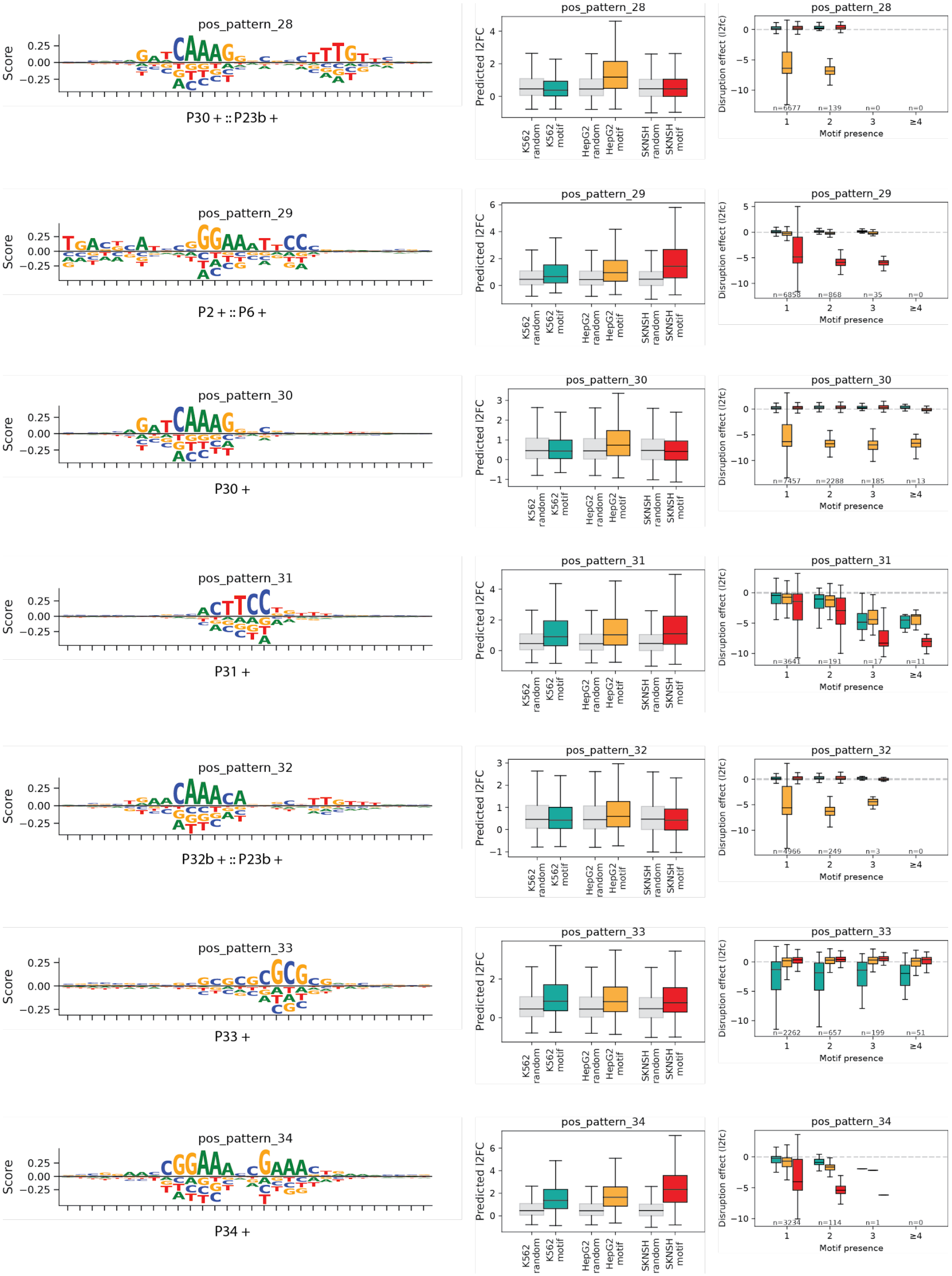
pos_pattern_28
P30 + :: P23b +
pos_pattern_29
P2 + :: P6 +
pos_pattern_30
P30 +
pos_pattern_31
P31 +
pos_pattern_32
P32b + :: P23b +
pos_pattern_33
P33 +
pos_pattern_34
P34 +
Score
Predicted l2FC
K562 random
K562 motif
HepG2 random
HepG2 motif
SKNSH random
SKNSH motif
Disruption effect (l2fc)
Motif presence

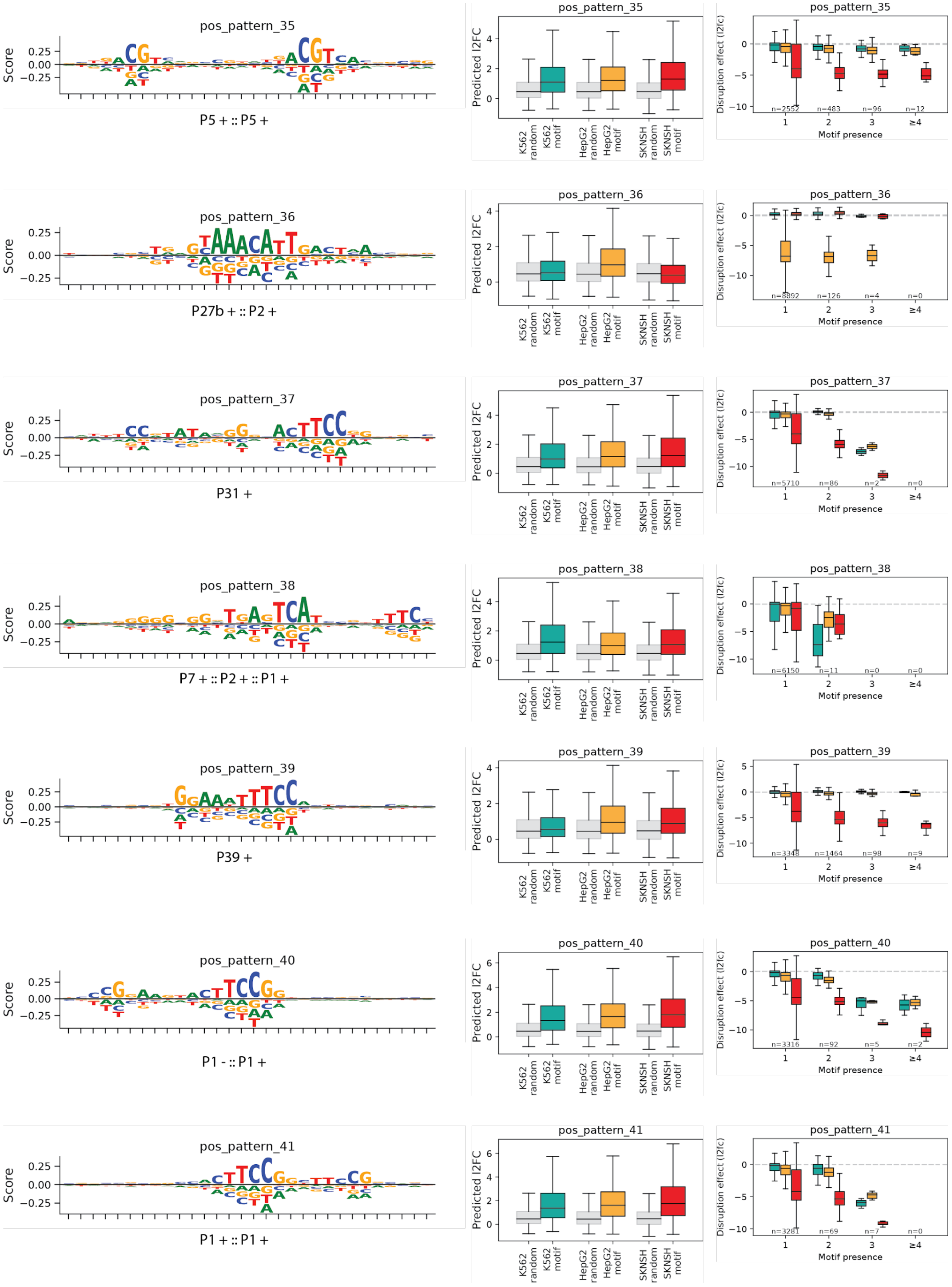

pos_pattern_35
Score
0.25
0.00
−0.25
P5 + :: P5 +
Predicted l2FC
K562 random
K562 motif
HepG2 random
HepG2 motif
SKNSH random
SKNSH motif
Disruption effect (l2fc)
n=2552
n=483
n=96
n=12
1
2
3
≥4
Motif presence
pos_pattern_36
P27b + :: P2 +
n=8892
n=126
n=4
n=0
pos_pattern_37
P31 +
n=5710
n=86
n=2
n=0
pos_pattern_38
P7 + :: P2 + :: P1 +
n=6150
n=11
n=0
n=0
pos_pattern_39
P39 +
n=3348
n=1464
n=98
n=9
pos_pattern_40
P1 - :: P1 +
n=3316
n=92
n=5
n=2
pos_pattern_41
P1 + :: P1 +
n=3281
n=69
n=7
n=0

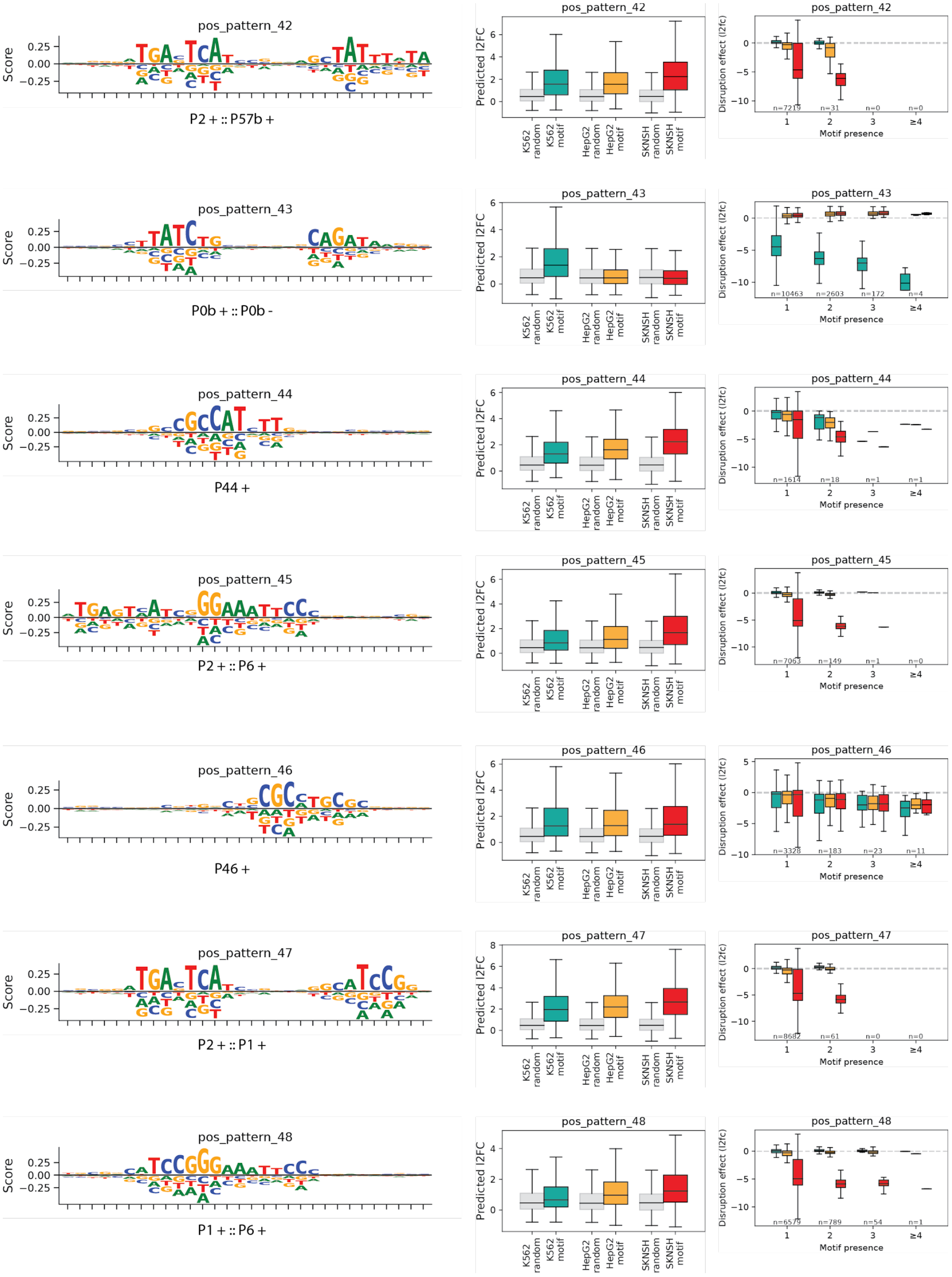

pos_pattern_42
P2 + :: P57b +
pos_pattern_43
P0b + :: P0b -
pos_pattern_44
P44 +
pos_pattern_45
P2 + :: P6 +
pos_pattern_46
P46 +
pos_pattern_47
P2 + :: P1 +
pos_pattern_48
P1 + :: P6 +
Score
Predicted l2FC
K562 random
K562 motif
HepG2 random
HepG2 motif
SKNSH random
SKNSH motif
Disruption effect (l2fc)
Motif presence
n=7219
n=31
n=0
n=0
n=10463
n=2603
n=172
n=4
n=1614
n=18
n=1
n=1
n=7063
n=149
n=1
n=0
n=3328
n=183
n=23
n=11
n=8682
n=61
n=0
n=0
n=6579
n=789
n=54
n=1

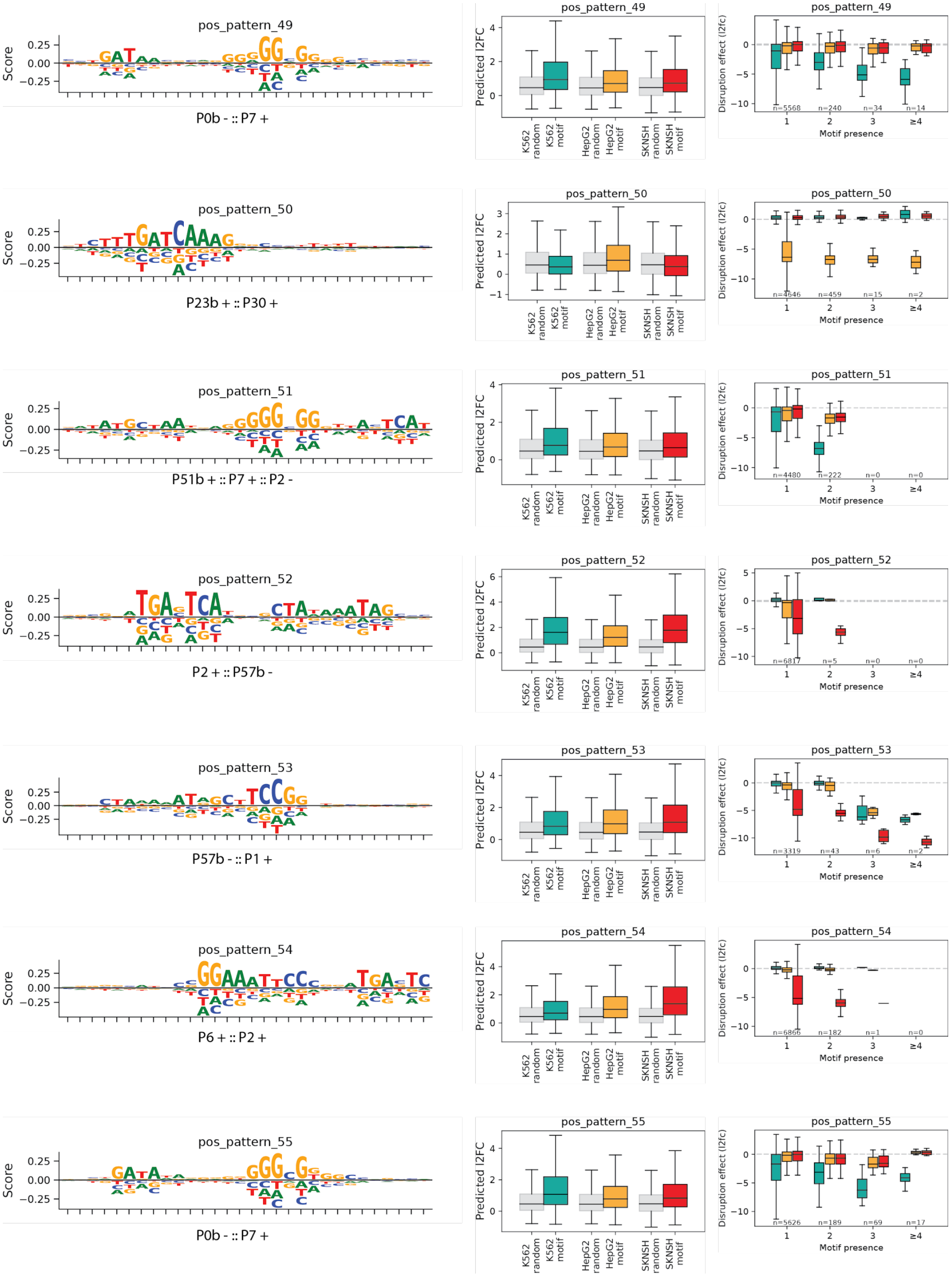
pos_pattern_49
Score
P0b - :: P7 +
Predicted l2FC
K562 random
K562 motif
HepG2 random
HepG2 motif
SKNSH random
SKNSH motif
Disruption effect (l2fc)
n=5568
n=240
n=34
n=14
Motif presence
pos_pattern_50
P23b + :: P30 +
n=4646
n=459
n=15
n=2
pos_pattern_51
P51b + :: P7 + :: P2 -
n=4480
n=222
n=0
n=0
pos_pattern_52
P2 + :: P57b -
n=6817
n=5
n=0
n=0
pos_pattern_53
P57b - :: P1 +
n=3319
n=43
n=6
n=2
pos_pattern_54
P6 + :: P2 +
n=6866
n=182
n=1
n=0
pos_pattern_55
P0b - :: P7 +
n=5626
n=189
n=69
n=17

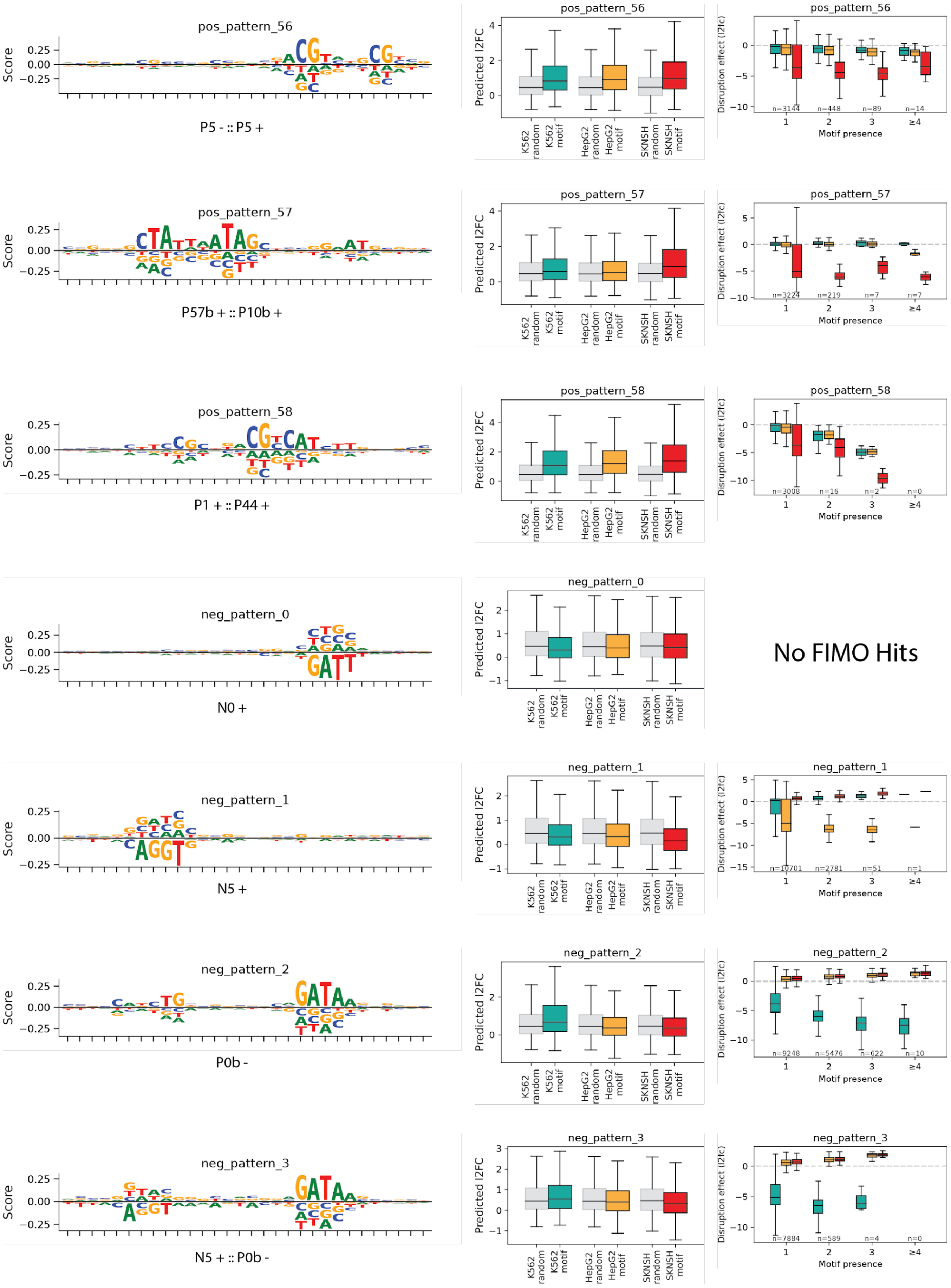
pos_pattern_56
P5 - :: P5 +
pos_pattern_57
P57b + :: P10b +
pos_pattern_58
P1 + :: P44 +
neg_pattern_0
N0 +
neg_pattern_1
N5 +
neg_pattern_2
P0b -
neg_pattern_3
N5 + :: P0b -
Score
Predicted l2FC
K562 random
K562 motif
HepG2 random
HepG2 motif
SKNSH random
SKNSH motif
Disruption effect (l2fc)
Motif presence
No FIMO Hits

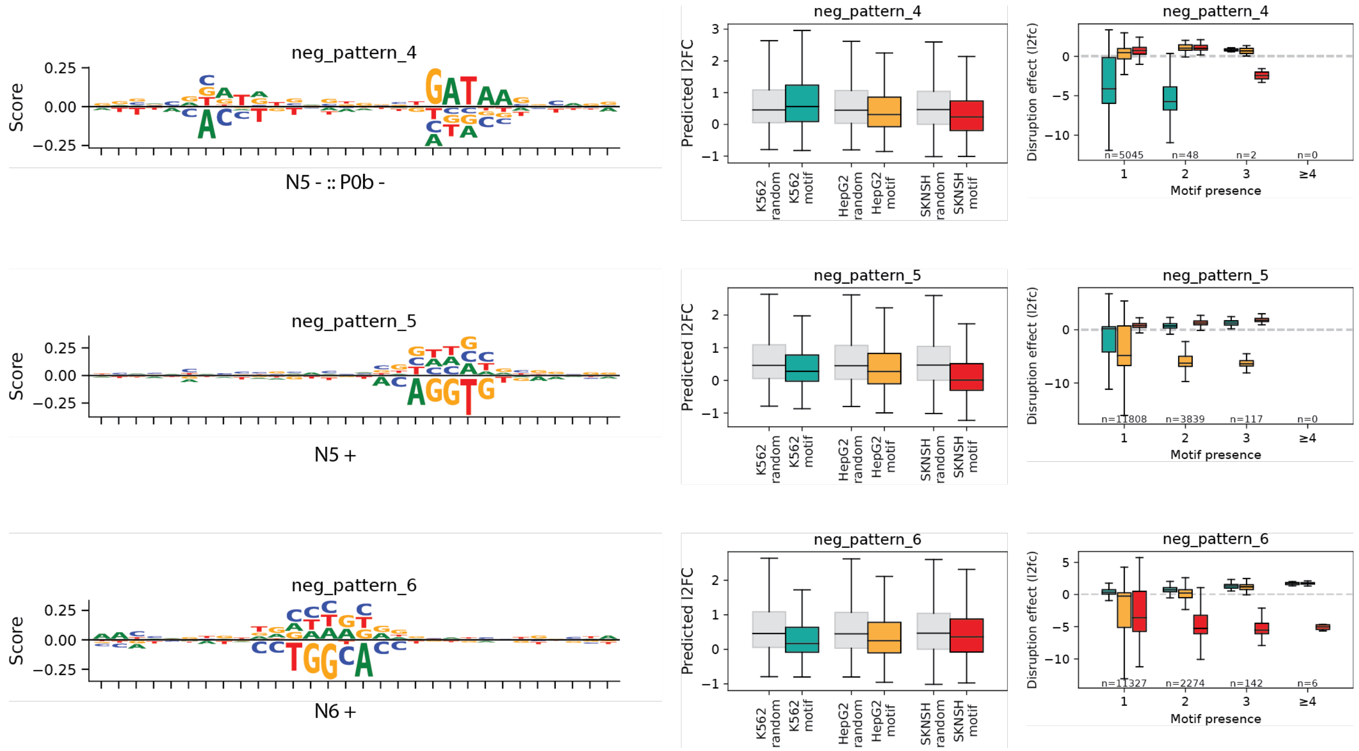

Supplementary Figure 12. Predicted functionality of TF-MoDISco original patterns. (**a**) Logos of the patterns found by TF-MoDISco. Names of core motifs forming the pattern are written below. The symbols +/- at the end of the name indicate the orientation of the match as forward or reverse complement respectively. (**c**) Activity predictions of sequences consisting of randomly sampled motif instances in the center and randomly background-sampled flanks in K562 (teal), HepG2 (gold), and SK-N-SH (red), along with activity predictions of fully random background-sampled sequences in K562, HepG2, and SK-N-SH (all in light gray). Boxes demarcate the 25th, 50th, and 75th percentile values, while whiskers indicate the outermost point within 1.5 times the interquartile range from the edges of the boxes. Number of sequences: n=5,000 for all boxes. (**d**) Predicted activity effect of disrupting all motif instances in the sequence library binned my motif presence score. Teal, gold, and red boxes correspond to effects to the predicted activity in K562, HepG2, and SK-N-SH, respectively. The *y*-axis corresponds to the activity prediction of the original (undisrupted) sequences minus the activity prediction of sequences with disrupted motif instances replaced by randomly background-sampled segments. The integer *n* below each bin of boxes indicates the number of sequences present in each motif score bin. Boxes demarcate the 25th, 50th, and 75th percentile values, while whiskers indicate the outermost point within 1.5 times the interquartile range from the edges of the boxes. Number of sequences is denoted below each set of boxes.

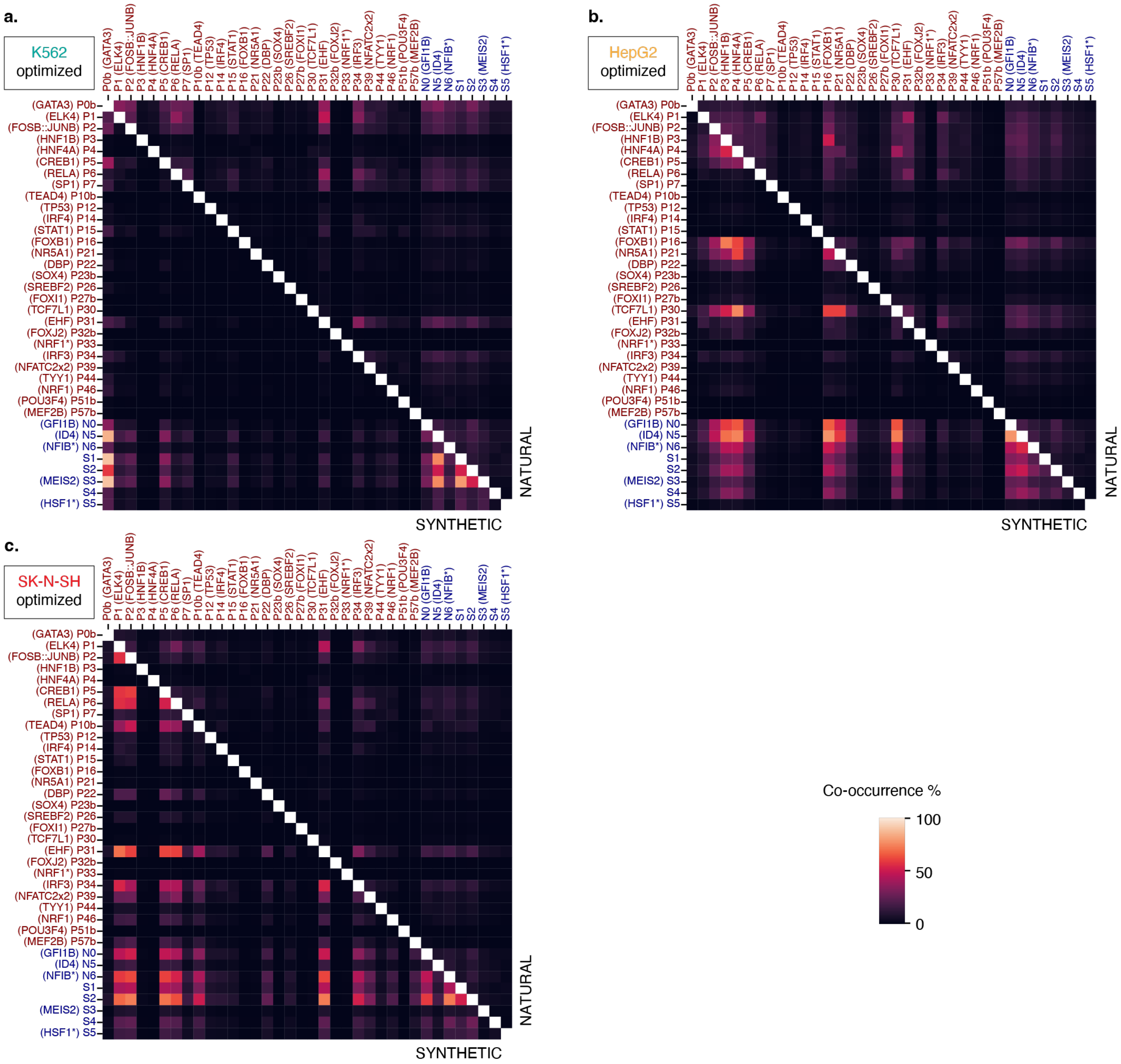

Supplementary Figure 13. Motif co-occurrence percentages. (**a**) Motif co-occurrence representation in K562-optimized sequences only. Color indicates the fraction of natural (upper triangle) or synthetic (lower triangle) K562-optimized sequences containing a motif pair. (**b**) Same as **a**, but in HepG2-optimized. (**c**) Same as **a**, but in SK-N-SH-optimized.

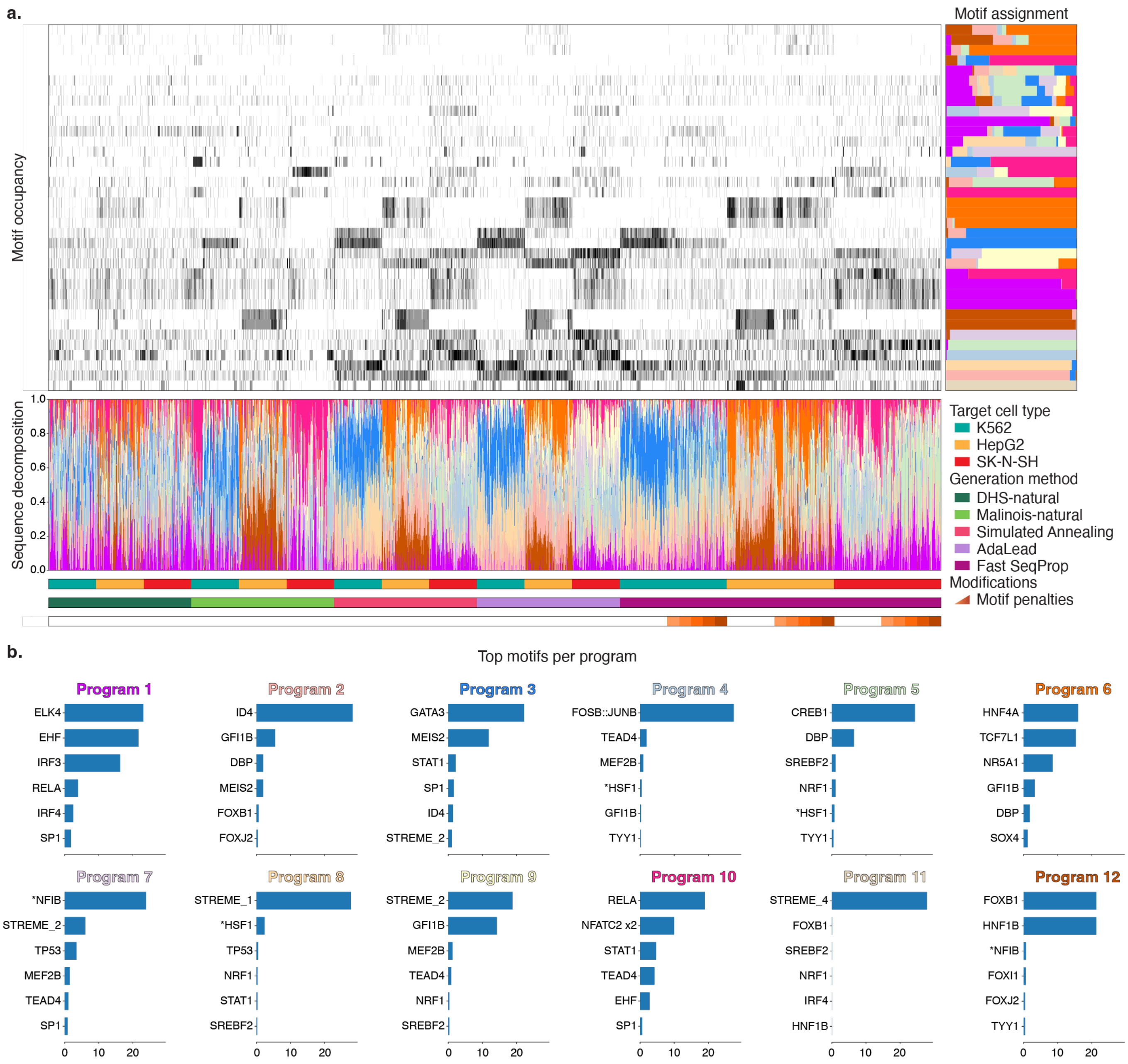

Supplementary Figure 14. Full NMF structure plot and top-motif set per program. (**a**) NMF decomposes sequence libraries and aggregates motifs into 12 distinct functional programs. Various CRE proposal methods favor distinct patterns of program usage. Top-left, grayscale heatmap: Motifs (*y*-axis) are identified in each sequence (*x*-axis). Shading indicates the number of motif matches in a sequence, capped at 5 matches. Top-right horizontal bar plot: Frequency of program association for each motif extracted from NMF feature matrix, unit normalized. *Y*-axis is shared with top-left and ordering was set by clustering motifs using the feature matrix. Program coloring is consistent with **Figure 3d**. Bottom, vertical bar plot: Program decomposition of individual sequences, unit normalized. Bottom, colored stips: Demarcation of CRE metadata (i.e., predicted target cell type, generation method, objective function modification) with color corresponding to legend on the right and side. CREs are clustered within these subsets based on program content. (**b**) Raw values from the NMF feature matrix for the top 6 motifs associated with each program. Coloring of program subtitles is consistent with **Figure 3d**.

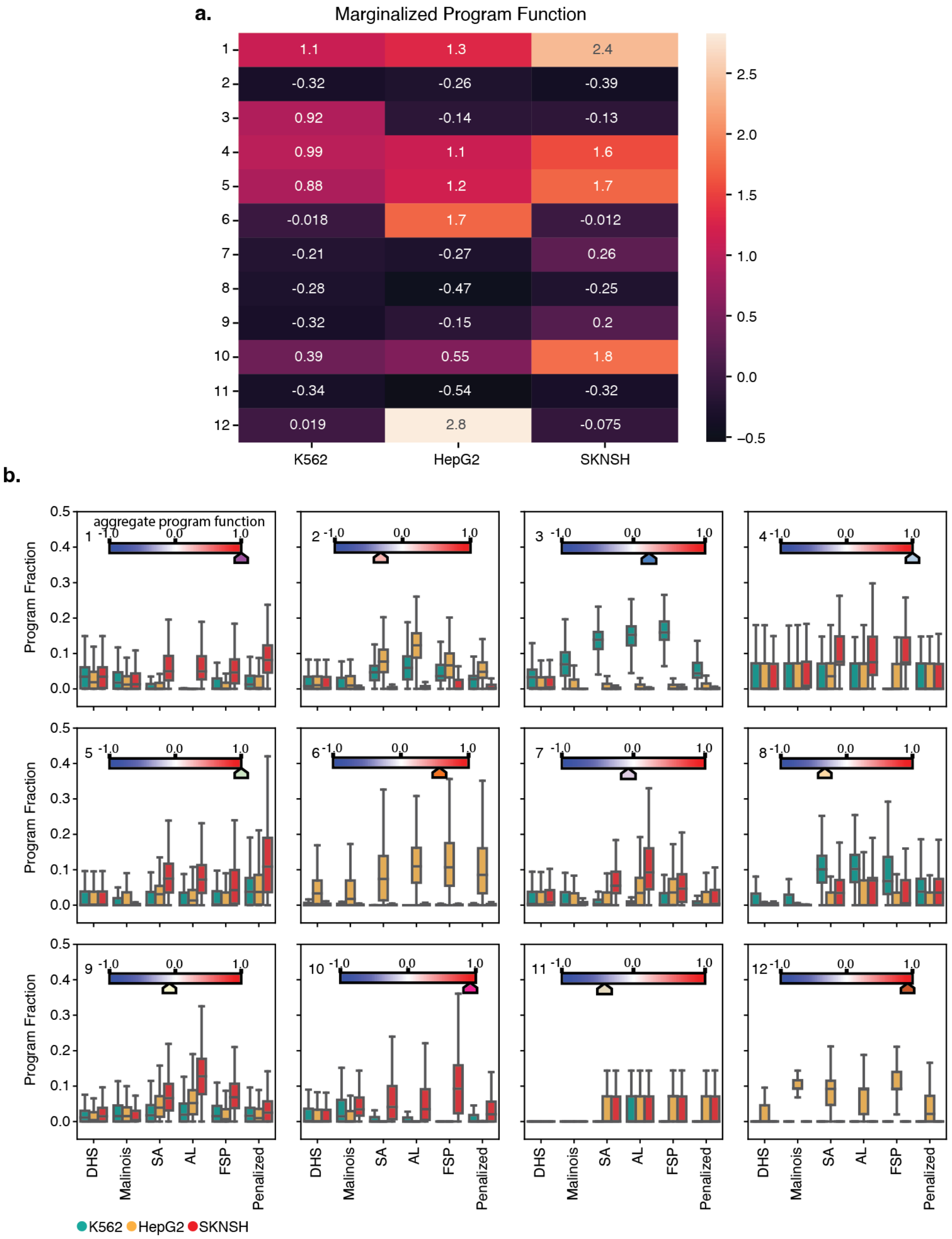

Supplementary Figure 15. Activating, repressing, and ubiquitous program content and usage. (**a**) Marginalized function of each NMF program in each cell type used to generate **Figure 3d**. These functional summaries are calculated using a weighted average of motif contributions (**Figure 3b**, **Methods**: Motif contributions) calculated using the unit normalized feature matrix from NMF (**Methods**). (**b**) Distribution of individual program fraction, normalized by total program content for 12 programs assessed by NMF decomposition. Sequences are grouped by design methodology (x-axis) and intended target cell type (hue). Inset slider indicates aggregate program function over K562, HepG2, and SK-N-SH (average repressive function indicated by blue, averages clipped within +/-1 range). Boxes demarcate the 25th, 50th, and 75th percentile values, while whiskers indicate the outermost point within 1.5 times the interquartile range from the edges of the boxes (n=4,000 for all boxes except for penalized where n=5,000).

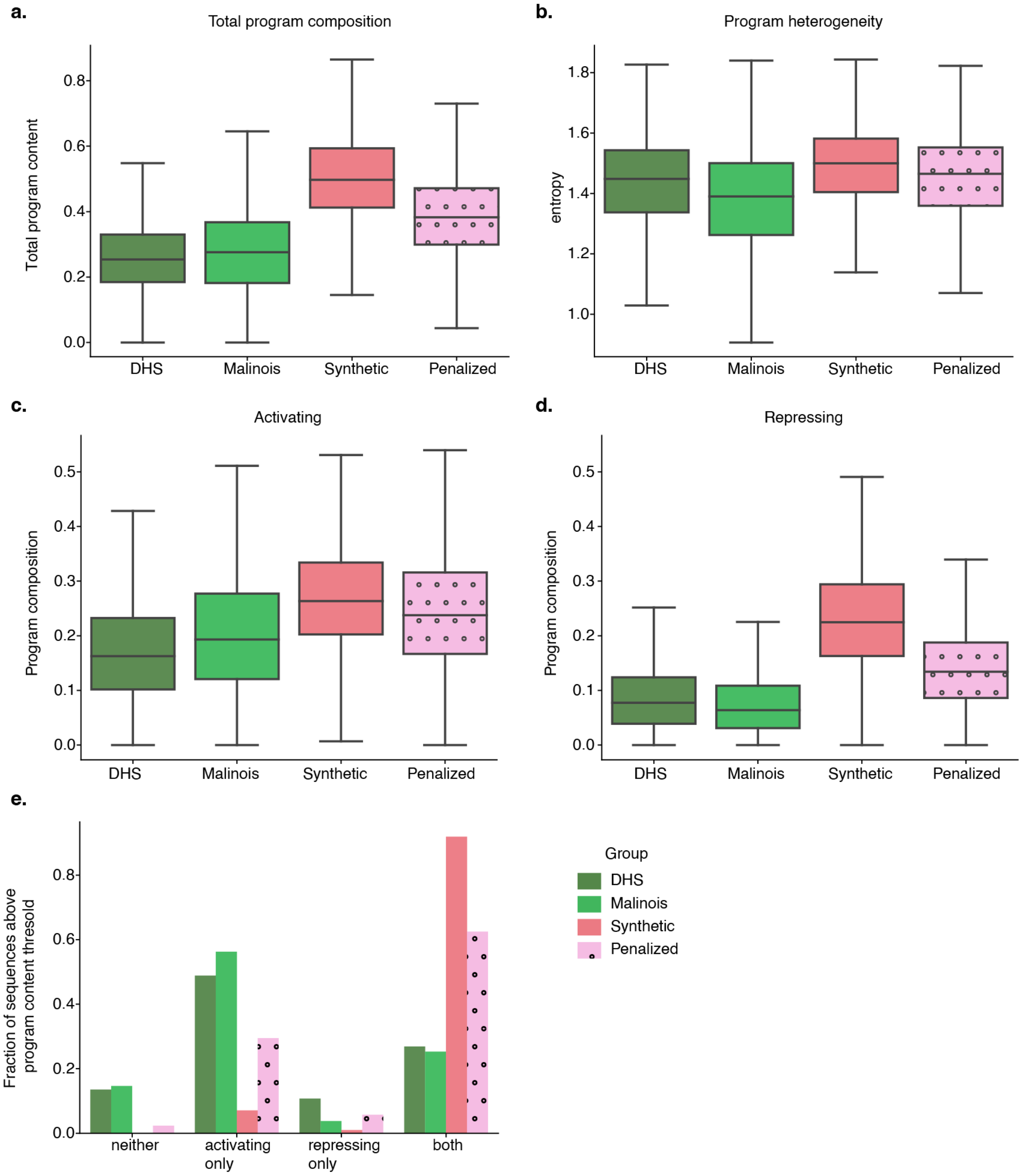

Supplementary Figure 16. Overall program usage. (**a**) Distribution of total program coefficients for sequences in different design groups, indicating the total amount of information encoded in each element. (**b**) Heterogeneity of program coefficients for each sequence measured by entropy. Higher entropy suggests greater diversity of programs used in each CRE. (**c**) Aggregating activating program content corresponding to the correct target cell type. High values indicate a greater proportion of information encoded in the CREs is dedicated to enhancing transcription in the target cell. (**d**) Same as c, except repressing programs. Higher values indicate a greater proportion of information encoded in the CREs is dedicated to repressing transcription in off-target cells. (a)-(d) Boxes demarcate the 25th, 50th, and 75th percentile values, while whiskers indicate the outermost point within 1.5 times the interquartile range from the edges of the boxes, outliers are indicated as points (n=12,000; 12,000; 36,000; 15,000 for DHS, Malinois, Synthetic, and Penalized, respectively). (**e**) Simultaneous usage of activating and repressing programs and motifs is the favored strategy for synthetic sequence design. Sequences are annotated as activating if composed of at least 1/10^{ths} activating programs and are annotated as repressing if composed of similar repressing program content. The fraction of sequences in each group passing none, strictly one, or both of these criteria are plotted.

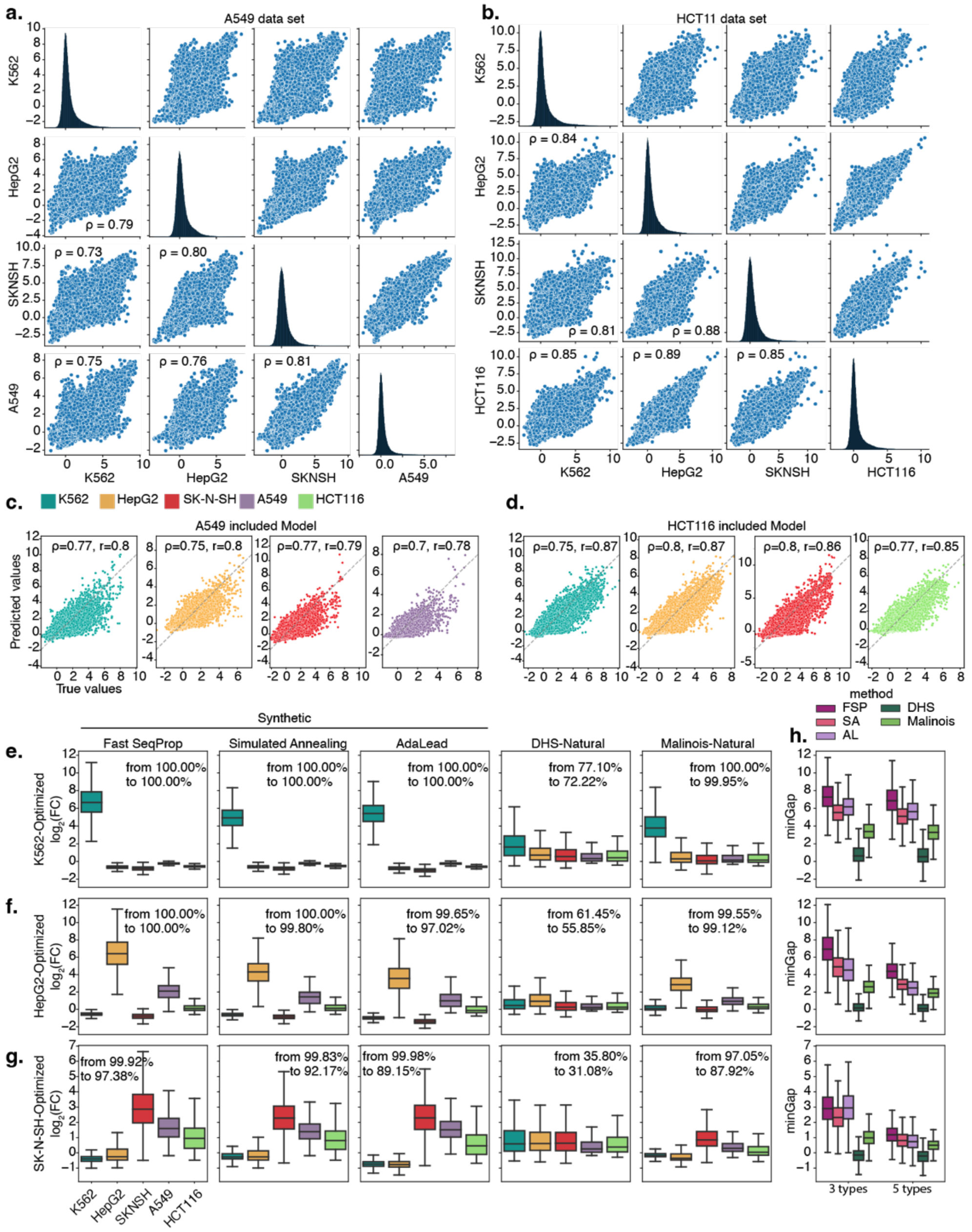

Supplementary Figure 17. MPRA models for A549 and HCT116 predict synthetic CREs. Additional MPRA measurements were made in A549 and HCT116 for 318,247 and 442,482 elements and used to model CRE activity in these cell lines, respectively. (**a-b**) Pairplot showing distribution of activity for sequences measured in (a) A549 and (b) HCT116 and other cell types. (**c-d**) A model trained on sequences with (c) A549 and (d) HCT116 measurements with the same settings as Malinois accurately predicts MPRA measurements of CRE function. Scatterplots show model performance on held out test data. (**e**) Predicted activity of K562-targeting CREs across 5 cell lines. CREs are separated into frames based on design methodology. Text inset indicates percentage of CREs where the intended target had the highest prediction before and after A549 and HCT116 predictions were considered. (**f**) Same as (e) except for HepG2-targeting CREs. (**g**) same as (e) and (f) except for SK-N-SH-targeting CREs. (**e-g**) Predictions made in 5 cell lines for n=4,000 elements in each subpanel. (**h**) On-target predicted activity of CREs summarized by minGap before and after A549 and HCT116 predictions were included in the calculation. Each frame collects CREs from the five frames to the left. Each box represents CREs from a different design method (n=4,000 elements per box). For each set of comparisons made using activity predictions for the same collection of cells, synthetic elements are predicted to maintain significantly higher average MinGap than any natural group both with and without A549 and HCT116 being considered in the calculations (p-adj<10^{-300} for all pairwise comparisons, Tukey's HSD test). (**e-h**) Boxes demarcate the 25th, 50th, and 75th percentile values, while whiskers indicate the outermost point within 1.5 times the interquartile range from the edges of the boxes.

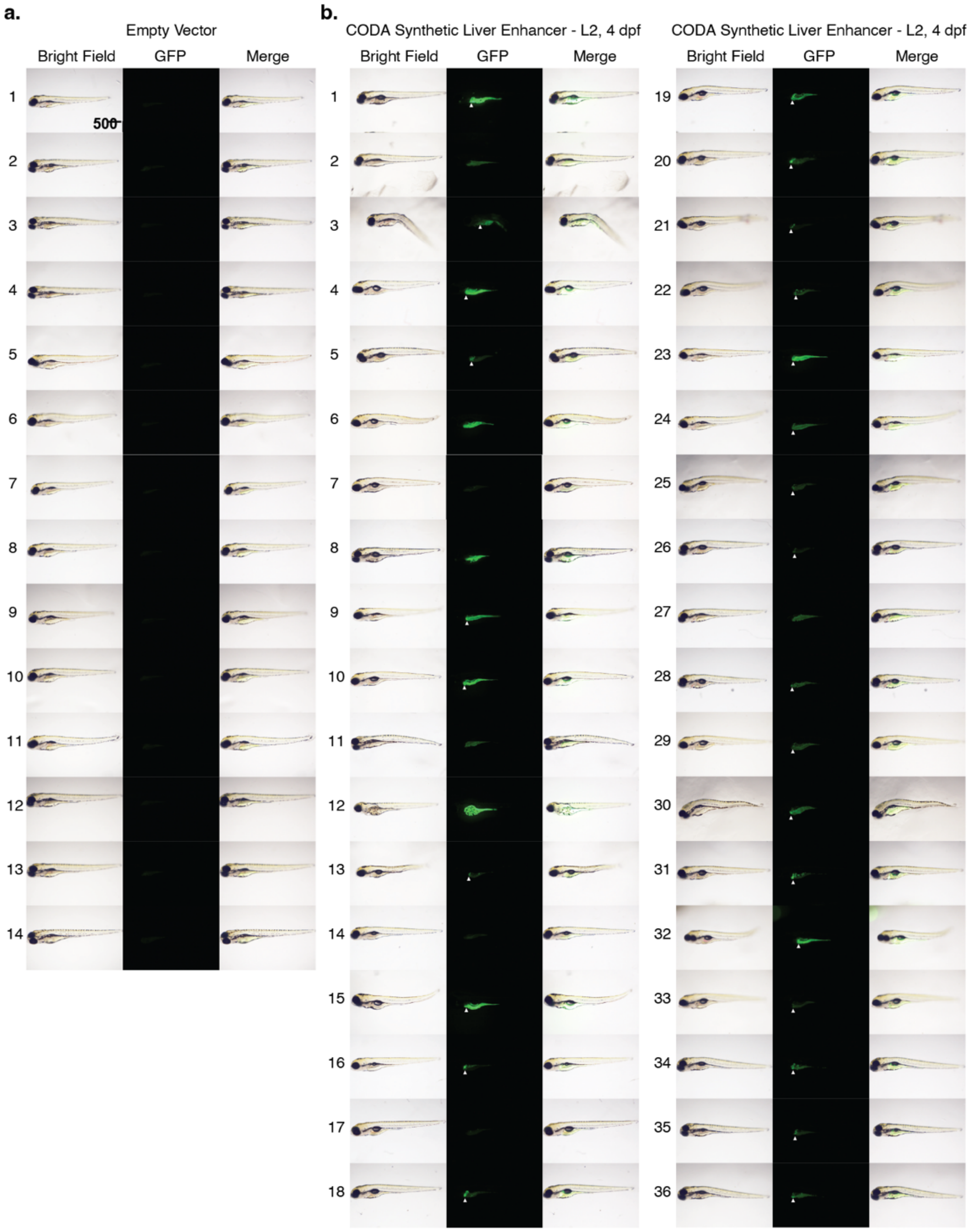

Supplementary Figure 18. A synthetic CRE reproducibly drives expression in zebrafish livers. (**a**) Expression of control transgene lacking synthetic CRE fails to drive GFP expression 4 days post-fertilization. All 14 control animals fail to show GFP expression. Faint signal is detected in multiple negative controls due to autofluorescence of the yolk sac. Animal 1 in this panel is duplicated from Figure 4b. (**b**) Synthetic CRE drives GFP expression in zebrafish livers and yolk-sacs. Synthetic CRE drives expression in zebrafish livers in 27 out of 36 animals, and yolk-sacs in 32 out of 36 animals. Animals 5, 16, 18, 34, and 35 in this panel are duplicated from Figure 4b.

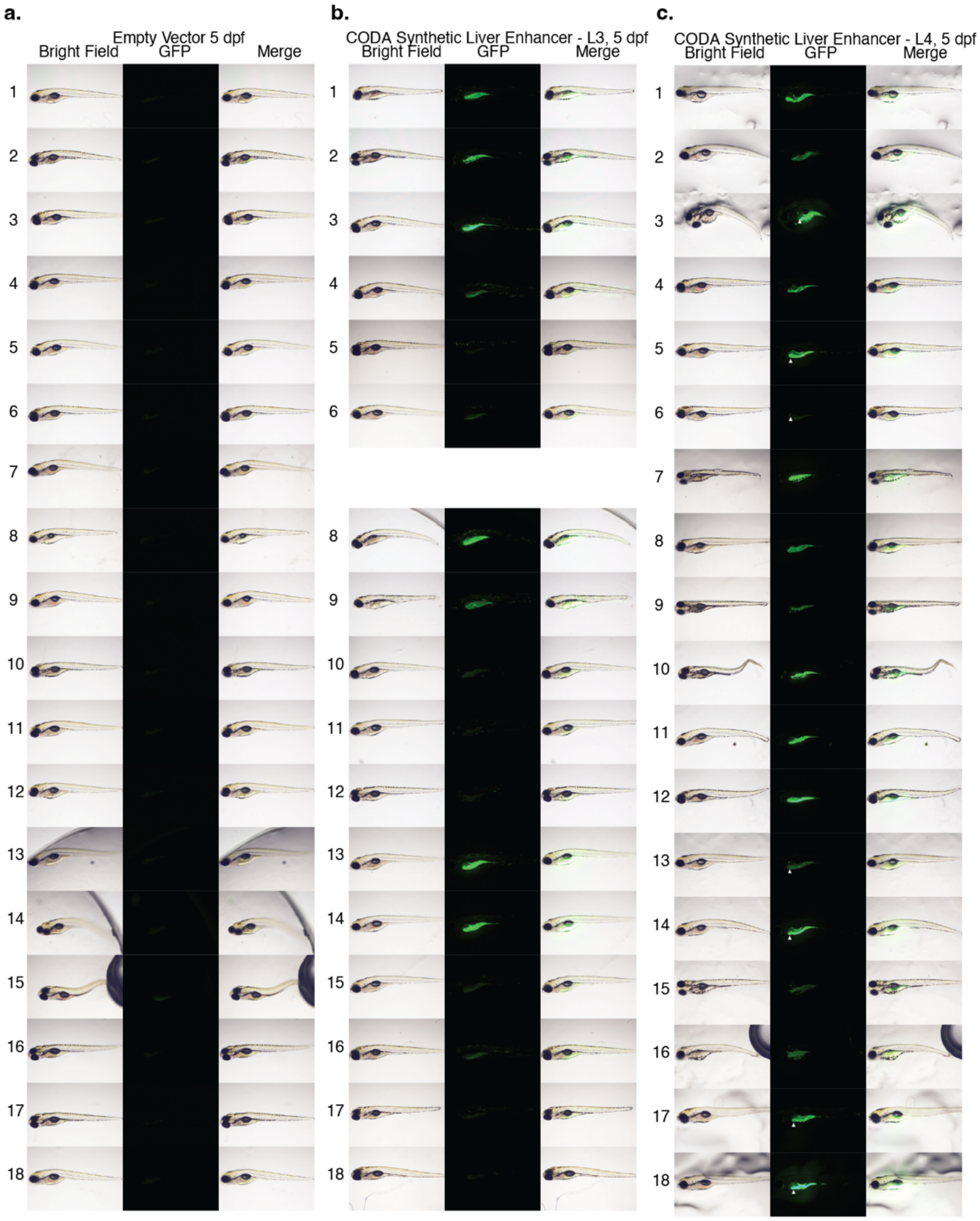

Supplementary Figure 19. Additional synthetic CREs drive expression in zebrafish gastrointestinal system. (**a**) Expression of control transgene lacking synthetic CRE fails to drive GFP expression 5 days post-fertilization. All 18 control animals fail to show GFP expression. Faint signal is detected in multiple negative controls due to autofluorescence of the yolk sac. (**b**) A second synthetic HepG2-specific CRE sporadically drives GFP expression in the yolk-sac, but not the liver. 8 out of 18 animals show CRE induced expression in the yolk-sacs 5 days post fertilization. (**c**) A third synthetic HepG2-specific CRE drives expression drives GFP expression in the liver and yolk-sac. White arrows indicate liver expression.

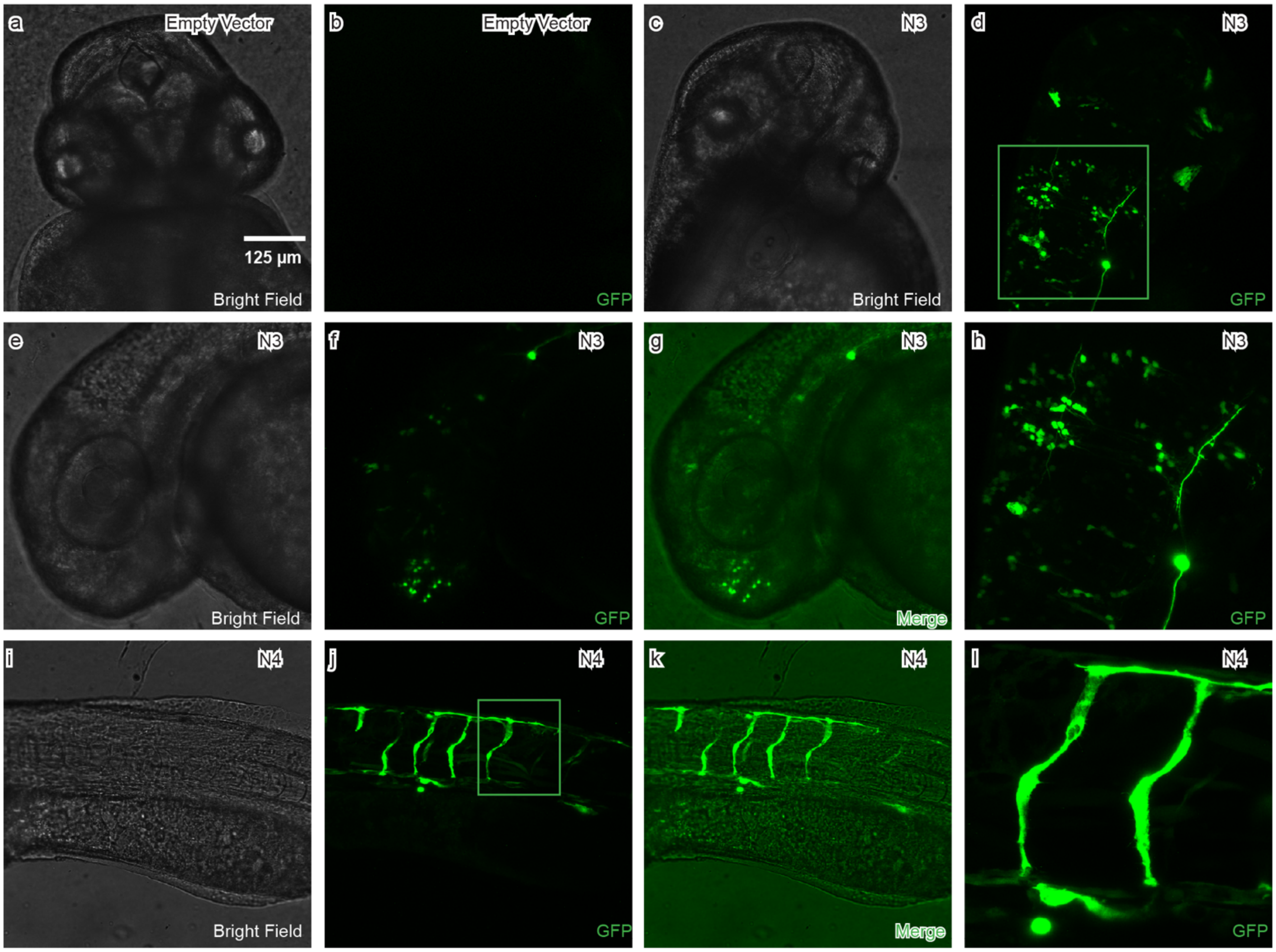

Supplementary Figure 20. SK-N-SH-specific CREs drive expression in zebrafish neurons or blood vessels. (**a**) Brightfield image of embryo 48 hours post-fertilization. (**b**) Control transgene lacking synthetic CRE fails to drive GFP expression in head of developing zebrafish. (**c**) Brightfield image of embryo transformed with transgene containing SK-N-SH-specific CRE (N3). (**d**) GFP channel of c. shows transgene expression in neurons. (**e**) Brightfield image of embryo transformed with transgene containing SK-N-SH-specific CRE. (**f**) GFP channel of **e** shows transgene expression in neurons. (**g**) Merged **e** and **f** (**h**) Zoom in of d. (**i**) Brightfield image of embryo transformed with another transgene containing SK-N-SH-specific CRE (N4). (**j**) N4 drives transgene expression in zebrafish blood vessel. (**k**) Merged **i** and **j**. (**l**) Zoom in of **j**. Panels **a**-**d**, **h**: Dorsal views, anterior top. Panels **e**-**g**, **i**-**l**: Anterior to the left, dorsal top.

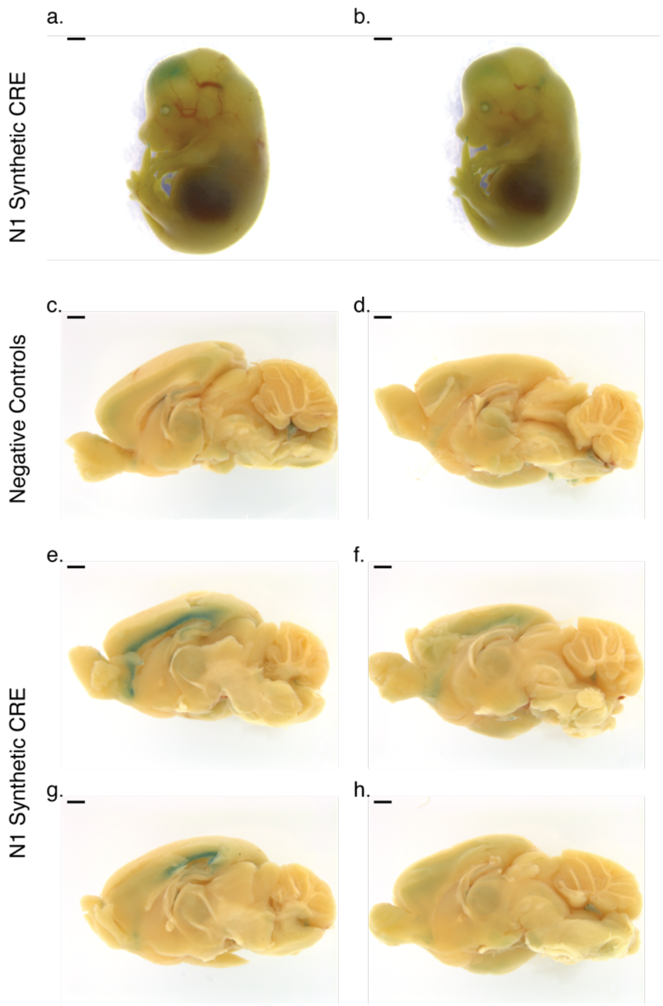

Supplementary Figure 21. Additional images from mouse transgenic experiments. (**a**) Synthetic neuronal CRE #1 (N1) and minP drive transgene expression in developing mouse forebrains. Day 14.5 mouse embryos whole animal lacZ staining. No control mouse. (**b**) Biological replicate of panel **a**. (**c**) Control brains without transgene drive minor transcriptional activation in 5 week old mice. Duplicated from **Figure 4d**. (**d**) Biological replicate of panel **c**. (**e**) N1 drives transgene expression cortical layer 6 in 5-week-old mouse brains in 3 out of 4 animals. This subpanel is duplicated from **Figure 4d**. (**f**) Biological replicate of panel **e**. (**g**) Biological replicate of panel **e**. Panels (e)-(f) represent all n=3 mice collected with cortical expression at 5 weeks postnatal. (**h**) Biological replicate of panel **e** that represents 1 of 3 mice without cortical transgene expression above the controls. (**a-h**) All scale bars: 1mm.

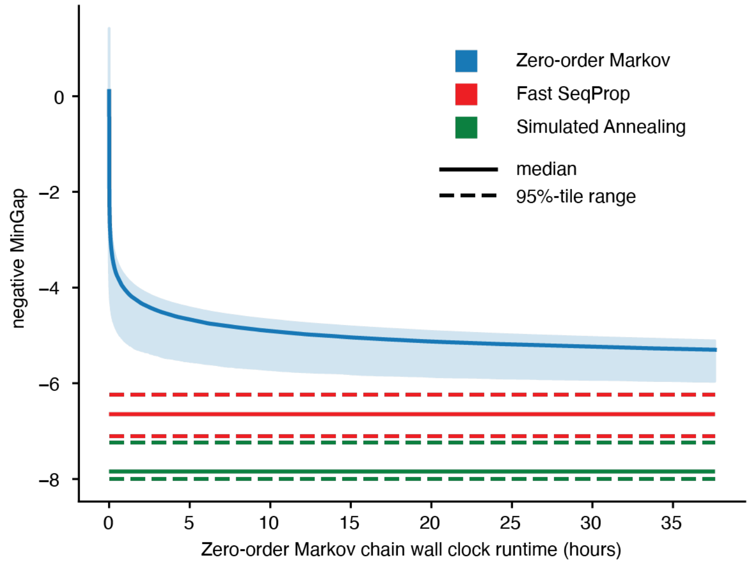

Supplementary Figure 22. Projection of efficiency of zero-order Markov chains for model directed sequence design. 200-mers were uniformly randomly sampled (i.e., sampled from a zero-order Markov chain) and tested using Malinois to calculate MinGap for K562 targeting sequences. We plotted the negative MinGap of the cumulatively best 15000 elements collected over 3000000 steps with 2048 samples taken at each step (total of 6.144 billion elements screened). We plot the median (blue line) and 95%-tile interval (blue shaded region) of the negative MinGap trajectory of the best element collection. As a comparison, we designed 15000 elements using Fast SeqProp (52.1 minutes) and Simulated Annealing (31.5 minutes) with the same objective and plotted the median and 95%-tile intervals of predicted MinGap for these groups.

Supplementary Tables Header Descriptions

Supplementary Table 1 Headers	Descriptions
Accession	Unique identifier within source database
Database	Name of the database used to source reference data set
Description	Short description of the data set
Link	URL used to access data set

Supplementary Table 2 Headers	Descriptions
IDs	Oligo IDs associated with the nucleotide sequence
chr	Chromosome
data_project	MPRA project
OL	Oligo library number(s) where the oligo was tested
class	trait category for UKBB, or chromatin mark for CRE (OL15)
K562_log2FC	average across oligo libraries (if applicable) of the mean across replicates of the log2(Fold Change) in K562
HepG2_log2FC	average across oligo libraries (if applicable) of the mean across replicates of the log2(Fold Change) in HepG2
SKNSH_log2FC	average across oligo libraries (if applicable) of the mean across replicates of the log2(Fold Change) in SKNSH
K562_lfcSE	log2FoldChange standar error in K562 (max across libraries if applicable)
HepG2_lfcSE	log2FoldChange standar error in HepG2 (max across libraries if applicable)
SKNSH_lfcSE	log2FoldChange standar error in SKNSH (max across libraries if applicable)
sequence	Nucleotide sequence

Supplementary Table 3 Headers	Descriptions
hepg2_test	test set performance for HepG2
hepg2_val	validation set performance for HepG2
sknsh_test	test set performance for SK-N-SH
sknsh_val	validation set performance for SK-N-SH
k562_test	test set performance for K562
k562_val	validation set performance for K562
batch_size	training loop batch size
padded_seq_len	total sequence length for model inputs after padding
duplication_cutoff	minimum activity cutoff for training set duplication
use_reverse_complements	training data augmentation, train on both forward and reverse complements of padded sequences
input_len	input length for model, should match padded_seq_len
conv1_channels	out_channels for torch.nn.Conv1d at the first layer
conv1_kernel_size	kernel_size for torch.nn.Conv1d at the first layer
conv2_channels	out_channels for torch.nn.Conv1d at the second layer
conv2_kernel_size	kernel_size for torch.nn.Conv1d at the second layer
conv3_channels	out_channels for torch.nn.Conv1d at the third layer
conv3_kernel_size	kernel_size for torch.nn.Conv1d at the third layer
n_linear_layers	number of fully connected layers folowing convolutional stack

linear_channels	out_channels for each fully connected layer folowing convolutional stack
linear_activation	activation function intervening fully connected layers
linear_dropout_p	dropout probability between fully connected linear layers
n_branched_layers	number of branched linear layers after fully connected stack and before output
branched_channels	number of output channels for each branch of the branched linear layers
branched_activation	activation function intervening branched linear layers
branched_dropout_p	dropout probability between branched linear layers
loss_criterion	loss function to use during training (see torch.nn.loss and custom loss functions in boda2)
parent_weights	path to pytorch state dict to initialze weights for transfer learning
frozen_epochs	number of epochs at the start of training where transfer learned weights are frozen
model_module	boda model module used for training
graph_module	boda graph module used for training
lr	learning rate
weight_decay	weight decay regularization
amsgrad	optimizer setting
T_0	scheduler argument
beta	loss funtion setting
betas	optimizer settings
timestamp	YYYYMMDD_HHMMSS timestamp

Supplementary Table 5 Headers	Descriptions
motif_id	motif ID (STREME format)
PWM	position-weight matrix
max_score	motif score of consensus sequence
target_cell	specificity target cell type
track_id	penalization track identificator
round	penalization round

Supplementary Table 6 Headers	Descriptions
File follows MEME motif format	https://meme-suite.org/meme/doc/meme-format.html

Supplementary Table 7 Headers	Descriptions
motif_id	motif ID as found in Sup Table 6 (Core motifs)
motif_alt_id	motif IDs for figure labeling; known TF motif match in parenthesis
sequence_name	sequence ID of motif hit
start	start of motif hit
stop	stop of motif hit
strand	strand orientation of motif hit
cell_type	cell type contribution score track of motif hit
pearson	hypothetical contribution score pearson correlation of motif hit

Supplementary Table 8 Headers	Descriptions
tissue	target tissue modeled using Enformer
selected_indices	indices selected to represent epigenetic and transcriptional signals of activation (Methods: Enformer analysis of epigenetic signatures)

Supplementary Table 9 Headers	Descriptions
module	module grouping for hyperparameters
hyperparameter	hyperparameter name
value	final value for hyperparameter
tuned	True if HPO was applied to this hparam during development

Supplementary Table 10 Headers	Descriptions
ID	oligo ID
sat_mut	allele ID: m{reference allele}{position}{alternate allele}
log2FoldChange	mean across replicates of the log2(Fold Change) in SKNSH
lfcSE	standar error of the log2(Fold Change) across replicates
celltype	cell type where MPRA was conducted

Supplementary Table 12 Headers	Descriptions
ID	sequence ID
sequence	nucleotide sequence
origin	method used to design/nominate the sequence
target_cell	specificity target cell type
round	penalization round (if applicable)
track_ID	penalization track identificator (if applicable)
K562_l2fc	mean across replicates of the log2(Fold Change) in K562
HepG2_l2fc	mean across replicates of the log2(Fold Change) in HepG2
SKNSH_l2fc	mean across replicates of the log2(Fold Change) in SKNSH
MinGap	log2FoldChange in target cell type minus the maximum log2FoldChange in off-target cell types
K562_lfcSE	standar error of the log2(Fold Change) across replicates in K562
HepG2_lfcSE	standar error of the log2(Fold Change) across replicates in HepG2
SKNSH_lfcSE	standar error of the log2(Fold Change) across replicates in SKNSH
K562_prediction	Malinois prediction in K562
HepG2_prediction	Malinois prediction in HepG2
SKNSH_prediction	Malinois prediction in SKNSH
