# Supplementary material for: Machine-guided design of cell-type-targeting cis-regulatory elements
Source: Nature. 2024 Oct 23;634(8036):1211–20. doi: 10.1038/s41586-024-08070-z (PMC11525185; doi:10.1038/s41586-024-08070-z)
Supplement: Supplementary file 2 — Reporting Summary [file 41586_2024_8070_MOESM2_ESM.pdf]

nature portfolio

Corresponding author(s): Ryan Tewhey
Steven K Reilly
Rodrigo I Castro
Sager J Gosai

Last updated by author(s): Aug 8, 2024

Reporting Summary

Nature Portfolio wishes to improve the reproducibility of the work that we publish. This form provides structure for consistency and transparency in reporting. For further information on Nature Portfolio policies, see our Editorial Policies and the Editorial Policy Checklist.

Statistics

For all statistical analyses, confirm that the following items are present in the figure legend, table legend, main text, or Methods section.

n/a	Confirmed	
☐	☒	The exact sample size (n) for each experimental group/condition, given as a discrete number and unit of measurement
☐	☒	A statement on whether measurements were taken from distinct samples or whether the same sample was measured repeatedly
☐	☒	The statistical test(s) used AND whether they are one- or two-sided<br>*Only common tests should be described solely by name; describe more complex techniques in the Methods section.*
☒	☐	A description of all covariates tested
☐	☒	A description of any assumptions or corrections, such as tests of normality and adjustment for multiple comparisons
☐	☒	A full description of the statistical parameters including central tendency (e.g. means) or other basic estimates (e.g. regression coefficient) AND variation (e.g. standard deviation) or associated estimates of uncertainty (e.g. confidence intervals)
☐	☒	For null hypothesis testing, the test statistic (e.g. F, t, r) with confidence intervals, effect sizes, degrees of freedom and P value noted<br>*Give P values as exact values whenever suitable.*
☒	☐	For Bayesian analysis, information on the choice of priors and Markov chain Monte Carlo settings
☒	☐	For hierarchical and complex designs, identification of the appropriate level for tests and full reporting of outcomes
☐	☒	Estimates of effect sizes (e.g. Cohen's d, Pearson's r), indicating how they were calculated

Our web collection on statistics for biologists contains articles on many of the points above.

Software and code

Policy information about availability of computer code

Data collection	Reference data collection: downloaded data from respective databases with default versions of Internet browsers, wget 1.21.2, and curl 7.81.0. Zebrafish imaging: Leica Application Suite LAS X 3.5.5.19976, Olympus cellSens Standard 2.3 (Build 18987). Mouse whole organism and tissue imaging: Leica Application Suite X 3.7.5.24914, FIJI 2.11.0. Mouse IHC imaging: Leica Thunder Imager, Leica Stellaris 8, FIJI 2.11.0.
Data analysis	MPRA results were analyzed with: MPRAmodel (v1.0.1) and MPRAcount (v1.0.1). Statistical tests and topic modeling: MPRAmodel (v1.0.2), DESeq2 1.32.0, Scipy 1.10.1, Sklearn 1.2.2, Pandas 1.5.3, Biopython 1.81. DNA sequence function modeling was performed using the CODA python library (github.com/sjgosai/boda2). Docker images for interactive development and running CODA-based applications can be found at gcr.io/sabeti-encode/boda. RNA-seq analysis conducted with: STAR (v2.5.2b), picard MarkDuplicates (MIT, v3.1.1), featureCount (v2.0.6), DESeq2 (v1.32.0). Mouse IHC co-staining quantification analyzed with Prism (v10.1).

For manuscripts utilizing custom algorithms or software that are central to the research but not yet described in published literature, software must be made available to editors and reviewers. We strongly encourage code deposition in a community repository (e.g. GitHub). See the Nature Portfolio guidelines for submitting code & software for further information.

Data

Policy information about availability of data

All manuscripts must include a data availability statement. This statement should provide the following information, where applicable:

- Accession codes, unique identifiers, or web links for publicly available datasets
- A description of any restrictions on data availability
- For clinical datasets or third party data, please ensure that the statement adheres to our policy

Reference data sets collected from the ENCODE and Zoonomia databases that were used in this study are linked and annotated in Supplementary Table 1. GRCh38/hg38 and GRCm38/mm10 were used as reference genomes in this study. Processed MPRA data used to train Malinois is available in Supplementary Table 2. Processed MPRA data and Malinois predictions for the cell type-specific CRE library designed for this study are available in Supplementary Table 12. Sequencing reads for RNA-seq are available in NCBI GEO (PRJNA1075667). Raw data, processing notebooks, model weights, and immunofluorescence images are available at https://zenodo.org/records/10698014.

Research involving human participants, their data, or biological material

Policy information about studies with human participants or human data. See also policy information about sex, gender (identity/presentation), and sexual orientation and race, ethnicity and racism.

Reporting on sex and gender	N/A
Reporting on race, ethnicity, or other socially relevant groupings	N/A
Population characteristics	N/A
Recruitment	N/A
Ethics oversight	N/A

Note that full information on the approval of the study protocol must also be provided in the manuscript.

Field-specific reporting

Please select the one below that is the best fit for your research. If you are not sure, read the appropriate sections before making your selection.

☒ Life sciences ☐ Behavioural & social sciences ☐ Ecological, evolutionary & environmental sciences

For a reference copy of the document with all sections, see nature.com/documents/nr-reporting-summary-flat.pdf

Life sciences study design

All studies must disclose on these points even when the disclosure is negative.

Sample size	Our sample size for each group of similarly derived candidate DNA elements was set to a minimum of 1000 and a maximum of 4000 for in vitro . This was based on intuition that downstream group-to-group comparisons would be well powered at these sample sizes. We were limited by the maximum tractable size of a single MPRA library at the time the experiments were done.<br><br>Sample size for sequences used for in vivo validation are limited by available experimental capacity. Results are reported for all synthetic CREs undergoing in vivo validation, including examples which failed to validate as expected. All in vivo data shown should be taken as representative.
Data exclusions	Data were not excluded.
Replication	MPRA assays were conducted in triplicate. All replicates of MPRA were successful. Three HepG2 specific synthetic CREs were used to produce transgenic zebrafish and replicated liver expression in 27/36; 0/17; and 7/18 animals with an additional 0/32 control animal demonstrating no expression. Three synthetic SK-N-SH specific CREs were used to produce transgenic zebrafish and and replicated neural transgene expression in 3/3; 3/3; and 0/3 animals imaged, with an additional 0/3 control animals demonstrating no expression. Two synthetic SK-N-SH specific CREs were used to produce transgenic mice. Only 1/2 synthetic SK-N-SH generated neural expression in mice. The synthetic SK-N-SH specific CRE that generated neural expression in mice was found to drive transgene expression in 3/6 5-week-old mouse brains. Negative control mice demonstrated transgene expression in 0/5 5-week-old mouse brains.
Randomization	Individual cells and animals were chosen randomly for this study from their existing cultures or colonies. MPRA randomizes CREs transfected into individual cells. Control and experimental animals were randomly assigned before transformation. Each group was tested with a predetermined sample size of 3 liters and all samples were stained regardless of their genotype and sex.

	Candidate cell type-specific CREs were selected or generated algorithmically without human intervention once algorithms were deployed. Additionally synthetic sequence generation begins with sequence randomization.
Blinding	Embryos were harvested and stained blindly with respect to their genotype.

Reporting for specific materials, systems and methods

We require information from authors about some types of materials, experimental systems and methods used in many studies. Here, indicate whether each material, system or method listed is relevant to your study. If you are not sure if a list item applies to your research, read the appropriate section before selecting a response.

Materials & experimental systems

n/a	Involved in the study
☐	☒ Antibodies
☐	☒ Eukaryotic cell lines
☒	☐ Palaeontology and archaeology
☐	☒ Animals and other organisms
☒	☐ Clinical data
☒	☐ Dual use research of concern
☒	☐ Plants

Methods

n/a	Involved in the study
☒	☐ ChIP-seq
☒	☐ Flow cytometry
☒	☐ MRI-based neuroimaging

Antibodies

Antibodies used	mouse anti-NeuN (abcam ab104224), chicken anti-GFAP (OriGene Technologies TA309150), rabbit anti-Iba1 (abcam ab178846). Secondary antibodies used were Goat anti-mouse Alexa Flour 488 (ThermoFisher Scientific, AB_ 2534069), Goat anti-chicken Alexa Flour 568 (ThermoFisher Scientific, AB_ 2534098), Goat anti-rabbit Alexa fluor 568 (abcam, ab175471).
Validation	All antibodies used are available from commercial vendors. Below lists vendor validated applications, total number of publications and relevant citations listed on the suppliers' websites.<br><br>• Mouse anti-NeuN (abcam ab104224): Monoclonal. Suitable for ICC/IF, WB, IHC-P. 582 publications. (https://pubmed.ncbi.nlm.nih.gov/27325769/)<br>• chicken anti-GFAP (OriGene Technologies TA309150): Polyclonal. Suitable for IF, WB. 3 publications. (https://pubmed.ncbi.nlm.nih.gov/34157194/)<br>• Mouse anti-Iba1 (abcam ab178846) Recombinant monoclonal. Suitable for WB, ICC/IF, Flow Cyt (Intra), IHC-P. 390 publications. (https://pubmed.ncbi.nlm.nih.gov/29769726/)<br>• Goat anti-mouse Alexa Flour 488 (ThermoFisher Scientific, AB_ 2534069): Polyclonal secondary Suitable IHC, ICC/IF, Flow Cytometry, 86 publications (https://pubmed.ncbi.nlm.nih.gov/36450710/).<br>• Goat anti-chicken Alexa Flour 568 (ThermoFisher Scientific, AB_ 2534098): Polyclonal secondary. Suitable for WB, IHC, ICC/IF, flow cytometry, 3 publications (https://pubmed.ncbi.nlm.nih.gov/32290848/).<br>• Goat anti-rabbit Alexa fluor 568 (abcam, ab175471): Polyclonal. Suitable for ELISA, IHC-Fr, IHC-P, Flow Cyt, ICC/IF, 187 publications. (https://pubmed.ncbi.nlm.nih.gov/33503434/)

Eukaryotic cell lines

Policy information about cell lines and Sex and Gender in Research

Cell line source(s)	All cell lines were purchased from ATCC (atcc.org). This study used K562, HepG2, and SK-N-SH cell lines.
Authentication	All cell lines were acquired from ATCC, authenticated using genotyping and gene expression signatures, routinely.
Mycoplasma contamination	All cell lines are tested monthly for mycoplasma and other common contaminants by The Jackson Laboratory's Molecular Diagnostic Laboratory.
Commonly misidentified lines (See ICLAC register)	*Name any commonly misidentified cell lines used in the study and provide a rationale for their use.*

Animals and other research organisms

Policy information about studies involving animals; ARRIVE guidelines recommended for reporting animal research, and Sex and Gender in Research

Laboratory animals	Mouse: Species - mus musculus, Strain - FVB (JAX ID #001800), Age - embryo to 5 weeks post natal. Zebrafish: Species - Danio rerio, [AB wild-type strain], Age - embryos up to 5 days post fertilization. All mice were housed in duplexed pens containing five or less mice and a 12-hour light/dark cycle at 18-23°C with 40-60% humidity.

Wild animals	This study did not involve wild animals.
Reporting on sex	Sex was not considered in this study.
Field-collected samples	This study did not involve field-collected samples.
Ethics oversight	All zebrafish procedures were approved by the Yale University Institutional Animal Care and Use Committee (IACUC) (Protocol Number 2022-20274). All mouse procedures were performed in accordance with the National Institutes of Health Guide for the Care and Use of Laboratory Animals, and were approved by the Institutional Animal Care and Use Committees of The Jackson Laboratory (protocol number 18038).

Note that full information on the approval of the study protocol must also be provided in the manuscript.
